# Progress in Gene Editing Tools and Their Potential for Correcting Mutations Underlying Hearing and Vision Loss

**DOI:** 10.3389/fgeed.2021.737632

**Published:** 2021-10-28

**Authors:** Catherine Botto, Deniz Dalkara, Aziz El-Amraoui

**Affiliations:** ^1^ Sorbonne Université, INSERM, CNRS, Institut de la Vision, Paris, France; ^2^ Unit Progressive Sensory Disorders, Pathophysiology and Therapy, Institut Pasteur, Institut de l’Audition, Université de Paris, INSERM-UMRS1120, Paris, France

**Keywords:** gene editing, CRISPR/Cas9, inherited retinal degeneration (IRD), blindness, deafness (hearing loss), gene therapy, hair cells, retina

## Abstract

Blindness and deafness are the most frequent sensory disorders in humans. Whatever their cause — genetic, environmental, or due to toxic agents, or aging — the deterioration of these senses is often linked to irreversible damage to the light-sensing photoreceptor cells (blindness) and/or the mechanosensitive hair cells (deafness). Efforts are increasingly focused on preventing disease progression by correcting or replacing the blindness and deafness-causal pathogenic alleles. In recent years, gene replacement therapies for rare monogenic disorders of the retina have given positive results, leading to the marketing of the first gene therapy product for a form of childhood hereditary blindness. Promising results, with a partial restoration of auditory function, have also been reported in preclinical models of human deafness. Silencing approaches, including antisense oligonucleotides, adeno-associated virus (AAV)–mediated microRNA delivery, and genome-editing approaches have also been applied to various genetic forms of blindness and deafness The discovery of new DNA- and RNA-based CRISPR/Cas nucleases, and the new generations of base, prime, and RNA editors offers new possibilities for directly repairing point mutations and therapeutically restoring gene function. Thanks to easy access and immune-privilege status of self-contained compartments, the eye and the ear continue to be at the forefront of developing therapies for genetic diseases. Here, we review the ongoing applications and achievements of this new class of emerging therapeutics in the sensory organs of vision and hearing, highlighting the challenges ahead and the solutions to be overcome for their successful therapeutic application *in vivo*.

## Introduction

**FIGURE 1:**
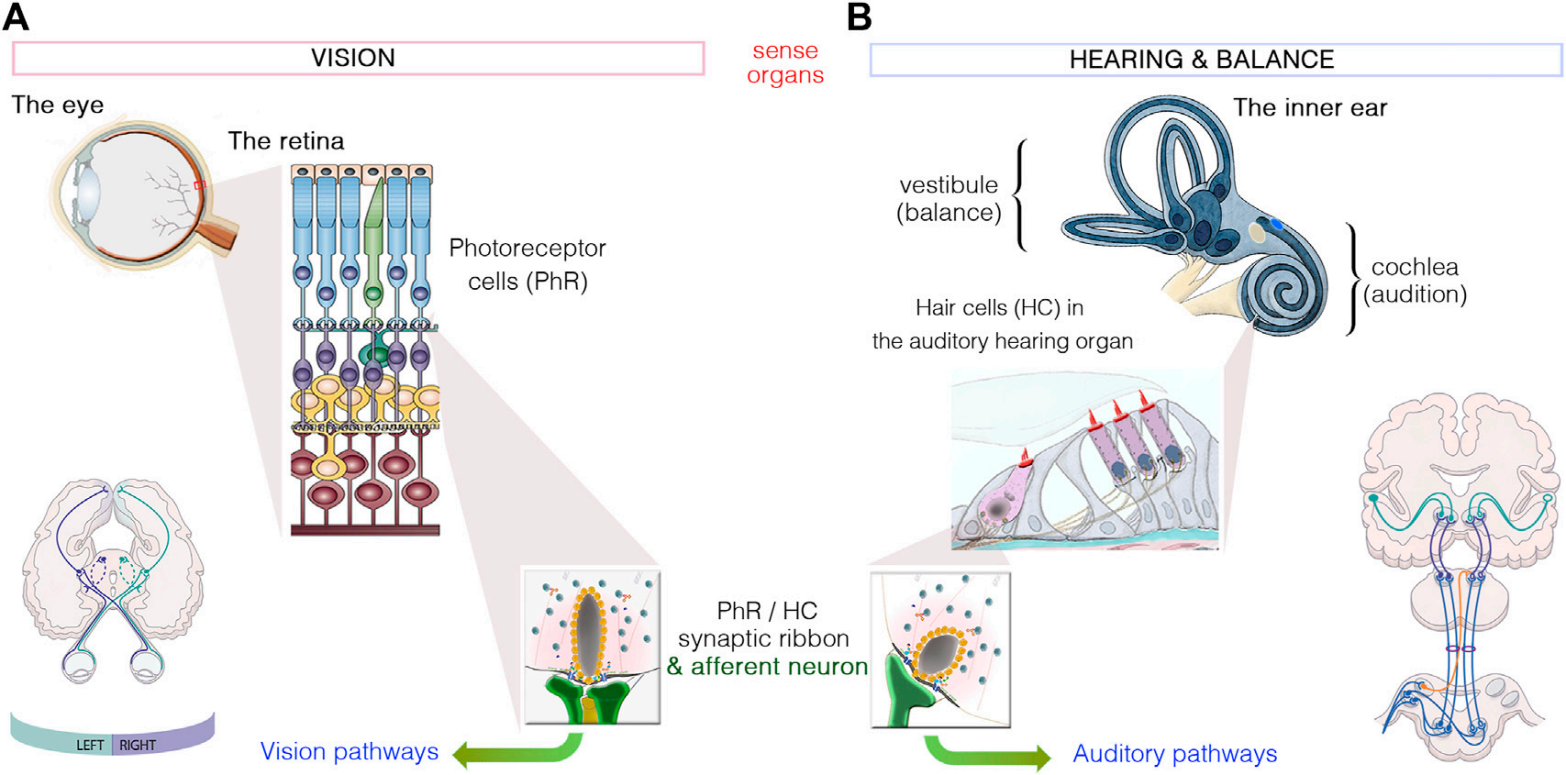
Similarities between the sensory organs of vision and hearing. **(A)**: Light signals are transduced by the outer segments of the photoreceptors, in the retina. **(B)**: Hearing and balance are dependent on the processing of sound waves and/or movement within the hair bundles, which crown hair cells located in the cochlea and vestibule of the inner ear, respectively. Photoreceptors and hair cells have synaptic active zones different from those of brain conventional synapses in that they are associated with an electron-dense ribbon surrounded by tethered synaptic vesicles. The neurotransmitter released from all photoreceptor cells and hair cells is glutamate, which creates the electrical signal conveyed by afferent neurons through either the visual or auditory pathways to the brain.

Communication is the essence of social interactions. Vision and hearing (Figure 1) are essential for every significant activity of daily life, from mobility and autonomy to an appreciation of music, art and nature. Any impairment of these senses has a profound negative impact on the quality of life of the affected individuals, restricting their communication and reducing access to social activities, entertainment and working opportunities. In turn, this can lead to social isolation and depression (Delmaghani and El-Amraoui, 2020; Géléoc and El-Amraoui, 2020; Crane et al., 2021). Based on comprehensive empirical data, the World Health Organization has estimated that about 285 million people worldwide currently suffer from severe visual impairment (see http://www.who.int/). Furthermore, about 466 million people worldwide (∼6% of the world population), including 34 million children, have a disabling hearing impairment (with or without balance deficits). This number is expected to increase to more than one billion people by 2050 (see http://www.who.int/). Together, these sensory deficits have a dramatic socio-economic impact on healthcare systems worldwide.

Through millions of years of evolution, each species has adapted to changes in its environment, to move, find prey or escape a predator, communicate and evolve to survive. Humans can perceive the glow of a candle at 27 km and hear the slight rustle of a leaf. We carry out these extraordinary tasks unconsciously, taking for granted a series of molecular, cellular and functional events enabling us to perceive and react to our environment. Our vision and hearing depend on highly specialized sensors that, when activated, communicate with billions of specialized neurons to process, decode, interpret and integrate the perceived messages, leading to appropriate behaviors. The structures of these two senses make them excellent models for studying the functioning of the central nervous system and for developing and validating new innovative therapeutic strategies for neurodegenerative diseases. Building on the accumulated knowledge about the disease mechanisms underlying vision and/or hearing loss, several treatment strategies, notably viral mediated gene replacement therapies, have been implemented to prevent or correct blindness and/or deafness phenotypes (Delmaghani and El-Amraoui, 2020; Botto et al., 2021). Also, recent advances in gene editing in sensory organs have provided a basis for potential one-shot treatments for vision and hearing loss, and these promising advances are likely to be at the forefront of further developments in gene editing to correct pathogenic mutations underlying hereditary diseases in the years to come. Here, we review progress in our understanding of hearing and vision disorders, and the development of tools for treating related sensory deficits, providing a snapshot of the current achievements and challenges to gene editing therapies in the eye and in the inner ear.

## How Do the Eye and the Inner Ear Work?

### Retinal Anatomy and Function

**FIGURE 2:**
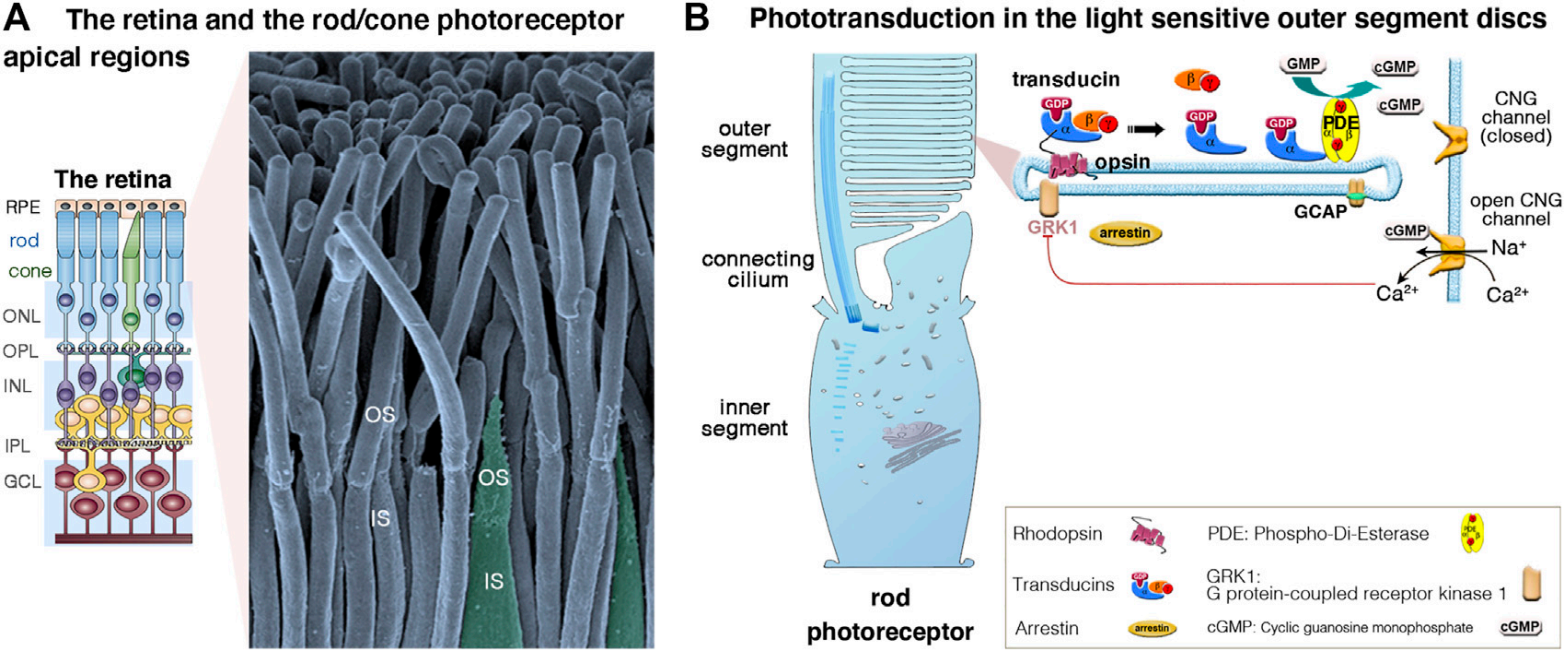
**(A**,**B)** The retina, photoreceptor cells and light-sensitive outer segments. **(A)** The mammalian retina, located at the back of the eye is a laminated, multilayer sensory epithelium, made up of the retinal pigment epithelium (RPE) attached to the different neuronal layers that compose the neuroretina: the outer nuclear layer (ONL), the inner nuclear layer (INL) and the ganglion cell layer (GC), separated by the synaptic Outer plexiform (OPL) and inner plexiform (IPL) layers. The scanning electron micrograph illustrates the apical functional compartments of the rod (nocturnal vision) and cone (diurnal vision) photoreceptor cells, the outer (OS) and the inner (IS) segments, separated by the connecting cilium. **(B)** Schematic representation of a rod apical region, illustrating the outer segment composed of hundreds to thousands of specialized membrane discs that house the phototransduction cascade machinery. Briefly, in absence of light, there is a constant influx of sodium and calcium ions across the cGMP-gated channels (CNGα1/β1) at the outer segment plasma membrane. Incoming light directly activates the light-sensitive opsins, rhodopsin (Rho, rods) and cone opsin, triggering the phototransduction process via the G protein transducins and the effector enzyme, cGMP phosphodiesterase (PDE). The increase of cytosolic cGMP leads to the closure of the CNG channels, photoreceptor cell hyperpolarization, and membrane potential changes leading to graded modulation of glutamate release at the photoreceptor synaptic terminals. Photoresponse is halted upon phosphorylation of opsins by opsin kinases (e.g., GRK1, rods; GRK7, cones) and by arrestin’s binding inactivating transducin.

The vertebrate retina (a 0.5 mm-thick tissue lining the back of the eye) consists of three layers of nerve cell bodies and two layers of synapses (Figure 2A) (Scholl et al., 2016; Malhotra et al., 2021). The outer nuclear layer contains the cell bodies of the rods and cones; the inner nuclear layer contains the cell bodies of the bipolar, horizontal and amacrine cells, and the ganglion cell layer contains the cell bodies of the ganglion cells and displaced amacrine cells. The photosensors (the rods and cones) lie outermost in the retina, against the retinal pigment epithelium (RPE), a layer of epithelial cells that nourish the photoreceptor cells, underneath the choroid. Light must, therefore, travel through the thickness of the retina to reach and activate the rods and cones (Figures 2A,B). Whilst rods are spread across retinal regions, cone cells display a greater concentration in the fovea (or “*area centralis*”), a unique structure only found in primates that allows high-acuity vision. Each cone synapses with one bipolar cell, creating high visual acuity but poor sensitivity in low light conditions. Several rods synapse with one bipolar cell, which greatly increase visual sensitivity in low light conditions. Under dark conditions, photoreceptors are constitutively in a depolarized state, thus continuously releasing glutamate across the synapse. When photons between 400 and 780 nm in wavelength enter the eye, they collide with the pigment molecules enriched within the light-sensitive outer segments of photoreceptors. Light stimulation of rhodopsin (in rods, which absorbs light with a wavelength of 500 nm), blue (440 nm)-, green (535 nm)- or red (565 nm) opsin in cones triggers the phototransduction biochemical cascade (Figure 2B), creating changes in electrical potentials of photoreceptor cells that is conveyed across all the inner retinal neurons of the retina (Figure 2A). Neural processing by these layers culminates in the inner plexiform layer, where the message concerning the visual image is transmitted to the brain as spike trains from the ganglion cells through the optic nerve (Figure 2A).

**FIGURE 3:**
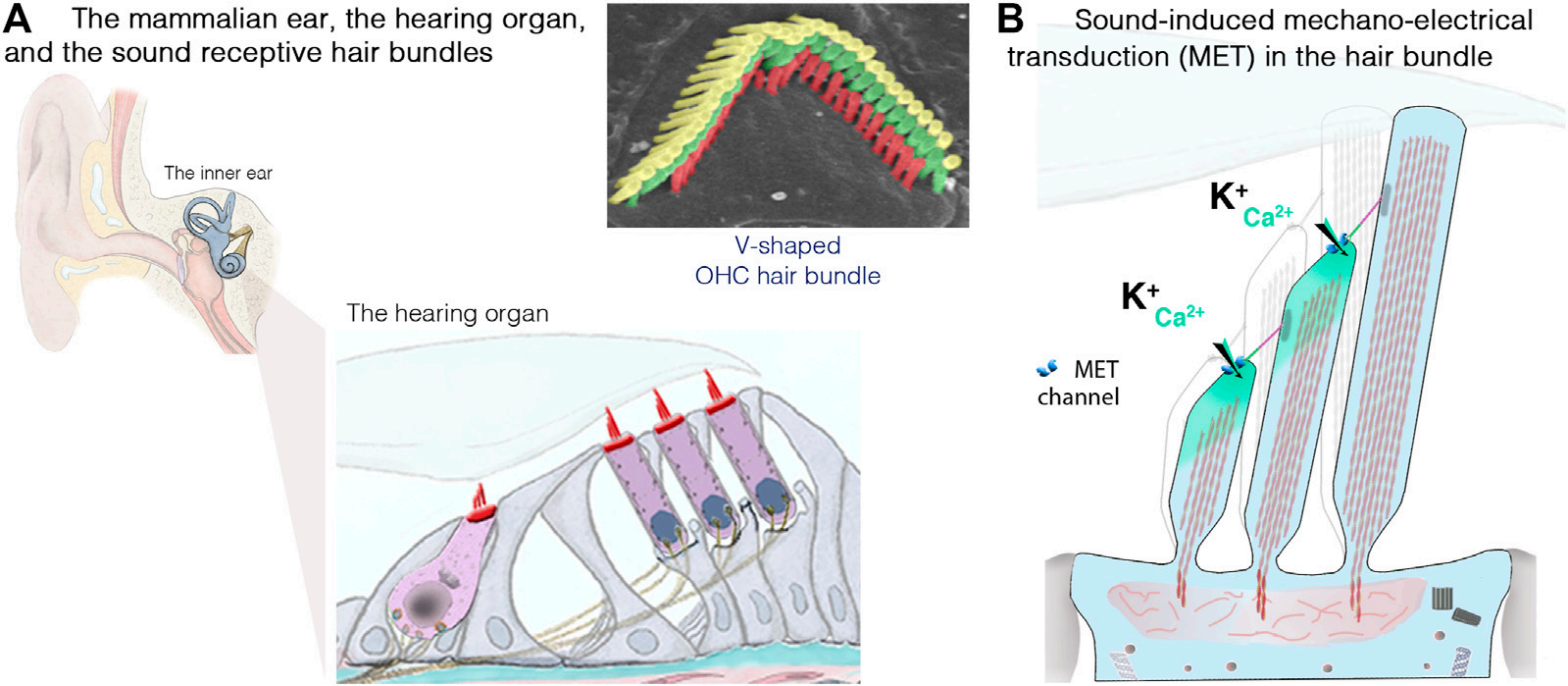
The inner ear, the hair cells and the sound-sensitive hair bundles. **(A)** The mammalian coiled, snail-shaped cochlea houses the auditory sensory organ, also called organ of Corti. Our hearing relies on two types of hair cells: a single row of inner hair cells (IHCs), the genuine sensory cells that transmit sound-induced electrical signals to the brain, and three rows of outer hair cells (OHCs), responsible for sound amplification. The scanning electron micrograph illustrate the highly organized structure of an OHC hair bundle, with three rows of stereocilia arranged in a staircase pattern. **(B)** Schematic representation of the mechanosensitive hair bundle, composed of 50–100 F-actin-filled microvillus structures, the stereocilia, arranged in staircase pattern at the apical hair cell surface. Incoming sound waves to the cochlea ultimately lead to the deflection of the hair bundle. Positive deflection, in the direction of the longest stereocilium, triggers the opening of the mechano-electrical transduction (MET) channels; located at the lower end of an extracellular fibrous link, the tip link. The ensuing influx of Ca^2+^ and K^+^ ions leads to hair-cell depolarization, resulting in membrane potential changes leading to graded modulation of glutamate release at the IHC synaptic active zones, which convey signal information to the brain through the auditory primary neurons (adapted from Dulon et al., 2018; Delmaghani and El-Amraoui, 2020).

Unlike the hair cells, which maintain throughout life their apical mechanosensitive hair bundles (Figures 3A,B), the photoreceptor cells renew their outer segments daily. Each day, approximately 10% of rod outer segment tips are shed and replaced with new disc formation at the outer segment base (Figures 2A,B). About 200 discs are replaced in each rod representing more than 20,000 μm^2^ of new membrane added at the outer segment base, and digested by the RPE, each day (reviewed in Malhotra et al., 2021).

### Inner Ear Anatomy and Function

The inner ear is home to the sensory organ for hearing, the cochlea, and the vestibular organs responsible for balance (Dulon et al., 2018; Delmaghani and El-Amraoui, 2020). Our ability to perceive sound and maintain balance depends on the process of mechanoelectrical transduction occurring in the mechanosensitive hair bundle, which crowns the apical region of the sensory auditory and vestibular hair cells of the inner ear. Each hair bundle consists of 50–300 F-actin-filled stereocilia, arranged in a highly organized staircase-like pattern (Figure 3A). Hearing is dependent on two types of hair cells in the cochlea: the outer hair cells (OHCs; 9,000–12,000 cells organized into four rows), which amplify sound stimuli, and the inner hair cells (IHCs; a single row of 3,000–3,500 cells), the genuine sensory cells responsible for transmitting sensory information to the central nervous system (Figure 3A). Upon deflection of the IHC hair bundle, cation influx through the mechanoelectrical transduction channels generates a depolarizing receptor potential (Figure 3B), which triggers Ca^2+^ influx through voltage-gated L-type channels in the presynaptic active zones, driving synaptic vesicle fusion and glutamate release by IHCs, and, thus, the transfer of acoustic information to the auditory neurons.

The sense of hearing developed early during evolution (Köppl and Manley, 2019; Delmaghani and El-Amraoui, 2020). An organ exclusively dedicated to hearing, the basilar papilla, first emerged in amphibians. The hearing organ gradually increased in size with evolution, eventually coiling to form the characteristics snail-shaped cochlea of mammals. Over millions of years of evolution, the hearing organ of each species has been optimized to perceive a specific range of frequencies. In humans, perceptible sound frequencies range from 20 Hz to 20 kHz. Our hearing and ability to discriminate speech depend on controlled spatial, tonotopic frequency organization in the cochlea, such that the physical, morphological and molecular properties of the hearing organ change gradually from the base to the apex of the cochlea. The base of the cochlea (where the sensory cells, particularly the OHCs, are shorter and more rigid) is dedicated to the analysis of high-frequency sounds, whereas the apex (where structures are thinner, with longer, more flexible cells) responds to low-frequency sounds (see Delmaghani and El-Amraoui, 2020; Safieddine et al., 2012).

## Inherited Retinal Dystrophies and Deafness

Vision and/or hearing impairments are the most common forms of sensory disorders in humans (Géléoc and El-Amraoui, 2020; Crane et al., 2021). Multiple causes can lead to sensory deficits, including genetic, environmental (including infections, oto- or photo-toxic drugs, noise or light exposure), or aging. Loss of either sense can occur at any age, and manifest as the sole symptom (isolated forms) or in association with other symptoms, as in the Usher syndrome (the leading cause of deaf-blindness in humans) (Bonnet and El-Amraoui, 2012; Géléoc and El-Amraoui, 2020). In developed countries, genetic causes account for over 50% of all cases of vision or hearing loss. The individual mutations or causal variants of each of the sensory defective genes are relatively rare, but, taken together, they are a significant cause of blindness and/or deafness, particularly in individuals of working age, which increases their economic and societal impact.

Inherited blindness and deafness are clinically and genetically heterogeneous. Retinal dystrophies are often characterized by progressive degeneration of the neuroretina and/or the RPE. More than 300 genes have been implicated in inherited vision impairments (RetNet: https://sph.uth.edu/RetNet/). Clinically, the forms of inherited retinal degeneration vary from early-onset macular degeneration, a progressive type of degeneration affecting central vision, to retinitis pigmentosa (RP) and Leber congenital amaurosis (LCA), which often manifest at birth and initially involves rod photoreceptors at the periphery of the retina. Among them, retinitis pigmentosa is the most common cause of blindness and leads first to night blindness with visual field restriction due to rod photoreceptors’ death then loss of central vision associated with cone photoreceptor degeneration. Inherited retinal degeneration may display autosomal-dominant, autosomal-recessive, or X-linked inheritance, and more complex or multifactorial inheritance patterns have also been reported, especially for some of the later-onset progressive diseases.

Like for retinal dystrophies, up to about 1,000 different genes are thought to be involved in hearing disorders (Ingham et al., 2019). Monogenic hearing impairment can be non-syndromic or syndromic. More than 150 non-syndromic deafness genes have been identified to date and many more remain to be discovered (Shearer et al., 1993; Azaiez et al., 2018; Delmaghani and El-Amraoui, 2020). Deafness genes are named according to their mode and inheritance and date of discovery, with DFNA indicating autosomal dominant inheritance, DFNB for autosomal recessive inheritance, DFNX or DFNY for X- and Y-linked forms, respectively and DFN for auditory neuropathies (see http://hereditaryhearingloss.org/).

## Gene Therapy for Inherited Retinal Dystrophies and Auditory Deficits

Besides progress in imaging technologies for clinical investigations (Rossi et al., 2011), increased efforts have been devoted to improve early genetic diagnosis for blindness and deafness disorders (Kremer, 2019; Thompson et al., 2020; de Bruijn et al., 2021). Current treatments for hearing loss are limited to hearing aids and cochlear implants, which partially restore hearing abilities (Müller and Barr-Gillespie, 2015; Kleinlogel et al., 2020). Retinal prosthesis and optogenetic strategies are being developed (Scholl et al., 2016; Ayton et al., 2020; Kleinlogel et al., 2020), some with promising success to restore sense perception in ongoing clinical trials (Sahel et al., 2021).

Over the past years, increasing efforts have been focused to develop treatment solutions to restore normal gene function by gene therapy in vision and hearing disorders (Delmaghani and El-Amraoui, 2020; Kleinlogel et al., 2020; Omichi et al., 2020; Maguire et al., 2021; Sahu et al., 2021). The first applications of gene therapy were focused on gene replacement (or gene supplementation) therapy where a healthy copy of a defective gene is brought into the cells bearing the mutation using viral vectors. Over 2 decades, at least five different adeno-associated virus (AAV)-*RPE65* products were tested using mouse and canine models of Leber congenital amaurosis 2 (LCA2), which led to a total of 13 clinical trials (Maguire et al., 2021). This pioneering work led to the historical approval of the first gene therapy product, Luxturna, by the Food and Drug Administration (FDA) and European Medicines Agency (EMA) to treat Leber congenital amaurosis. This groundbreaking advancement of RPE65 gene therapy paved the way for numerous breakthrough clinical phase I/II trials exploring gene replacement for monogenic recessive diseases of the retina, such as choroideremia, retinoschisis, achromatopsia, Usher syndrome, and Leber hereditary optic neuropathy (Buck and Wijnholds, 2020; Botto et al., 2021; Crane et al., 2021). Thanks to new generation of viral vectors, successful gene replacement strategies targeting key deafness genes have been established, using single or double AAV vector-mediated delivery (see Sacheli et al., 2013; Askew and Chien, 2020; Géléoc and El-Amraoui, 2020). Almost all the genes studied are expressed in the hair cells, and they include the genes defective in Usher syndrome, such as the harmonin (USH1C) (Pan et al., 2017), sans (USH1G) (Emptoz et al., 2017), clarin-1 (USH3A) (Geng et al., 2017; Dulon et al., 2018; György et al., 2019a), whirlin (USH2D) (Isgrig et al., 2017) genes, and the genes encoding SYNE4 (Taiber et al., 2021), the mechano-electrical transduction channel proteins TMC1/2 (Wu et al., 2021; Nist-Lund et al., 2019), and the synaptic proteins VGLUT3 (Akil et al., 2012) and otoferlin (Akil et al., 2019; Al-Moyed et al., 2019). Several attempts have also been made to develop gene replacement strategies for the gap-junction protein connexin-26 (GJB2) (Iizuka et al., 2015), defects of which cause one of the most common forms of deafness (30–50% of recessive forms of profound deafness in children from Mediterranean countries). These efforts have benefited from viral vectors that efficiently target inner ear supporting cells (Askew and Chien, 2020; Zhang et al., 2020a).

The multiple causes, the diversity of cellular targets and pathways, along with great diversity of severity and progression rates of the sensory impairments are all key elements to integrate into the design of a therapeutic approach. Alongside gene replacement strategies, RNA-based therapies [e.g., antisense oligonucleotides (ASOs), RNA interference (RNAi), or read through agents] have been developed to silence the messenger RNA (mRNA) transcribed from the mutant allele by Watson-Crick base pairing (Maeda et al., 2009). For instance, the ASO approach has been successfully used to bypass or correct blindness/deafness causal mutations, leading to recovery of normal hearing and balance function (e.g., USH1C, Lentz et al., 2020) or retinal function (e.g., USH2A, Dulla et al., 2021). A phase 1/2 clinical trial (NCT03780257) uses an ASO designed to trigger skipping of exon 13 in USH2A, enabling expression of a functional protein. The ASO, QR-421a, was well tolerated, with a concordant benefit in multiple measures of vision (https://www.proqr.com/QR-421a-for-USH2A-RP). These positive results fostered ongoing plans for a phase2/3 trial (NCT03780257), but probably using repeated injections of QR-421a to ensure long-lasting and stable beneficial outcomes for vision.

Over the past few decades, we have witnessed a steady progression of techniques and technologies that enable site specific programmable genome editing. Indeed, the early generations of genome editing tools including meganucleases, zinc-finger nucleases (ZFNs) and transcription activator like effector nucleases (TALENs) paved the way to the development of tools enabling *in situ* modification at specific region of the defective target gene (Zhang et al., 2020b; Hirakawa et al., 2020; Wu et al., 2020). The groundwork on these first DNA editors set the foundation for the emergence and applications of the recently developed clustered regularly interspaced short palindromic repeats (CRISPR)-driven gene-editing techniques, boosting studies of DNA- and RNA-sequence modifications (Anzalone et al., 2020; Saha et al., 2021). Thanks to their simple programming to target specific DNA regions, via easily designed RNA guides, the rapidly evolving CRISPR–Cas editors broadened the scope of therapies for genetic diseases (Hirakawa et al., 2020). By directly correcting the gene or causing a reversion of the disease-causing mutation, these gene editing tools provide new opportunities for effective and long-lasting treatment of both dominant and recessive forms of hearing loss, regardless of the size of the gene or the nature of the mutation (Anzalone et al., 2020; Hirakawa et al., 2020; Saha et al., 2021 and cf. below).

For all viral and non-viral gene therapies, the specific delivery to ocular or auditory target cells requires appropriate use of vectors and routes of administration to ensure safety, efficacy and specificity (Nyberg et al., 2019; Yu and Wu, 2021; Sahu et al., 2021). Here below, we detail cell-specific delivery attempts for auditory and ocular disorders.

## The Improvement of Delivery Technologies Drives Major Advances in Eye and Inner Ear Therapeutics

**FIGURE 4:**
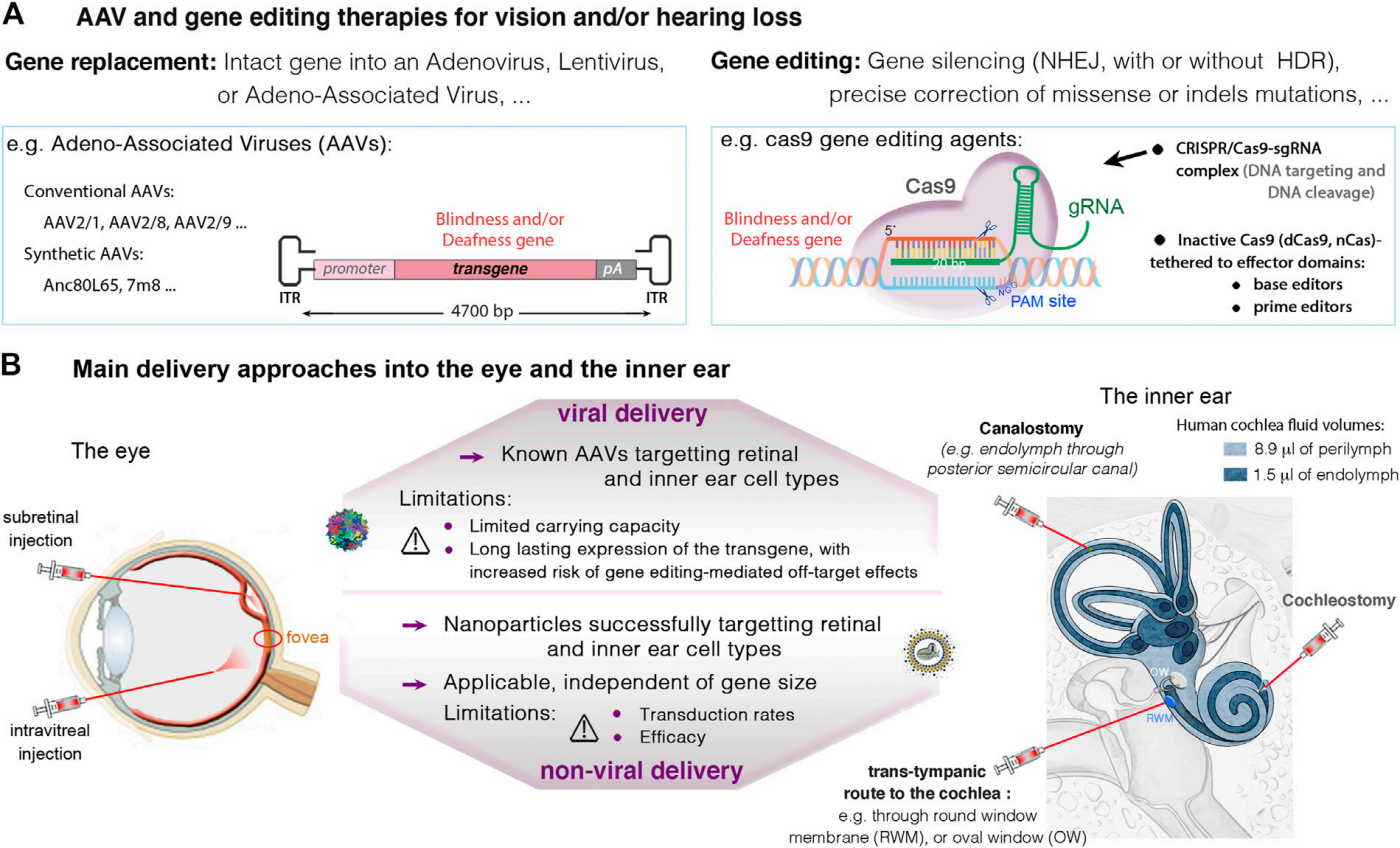
**(A)** Viral and non-viral delivery, and gene editing therapies in the eye and the inner ear. **(A)** Current therapeutic strategies with promising success in the eye and the inner ear involve gene replacement using adeno-associated viruses (AAVs), and gene editing targeting specific mutations causing vision and/or hearing loss. **(B)** An important challenge in eye and ear therapeutics is the route of administration. In the retina, the subretinal injection may be favored to ensure high transduction rates of photoreceptor and RPE cells. The intravitreal route is recommended for targeting the ganglion and inner nuclear cell layers, and particularly, the central retina. In the inner ear, main administration routes include local injections into the perilymph through the round-window membrane (RWM), or through the oval-window (trans-stapedial injection). Injections into the endolymph, in the cochlear scala media (cochleostomy) or through a semicircular canal (canalostomy), remain challenging, with high risk of sensory damage. Thanks to smaller size cas9 enzymes, it’s possible to use AAVs to transfer the CRISPR-Cas9/gRNA complex into target cells. Alternatives exist for larger nucleases such as base or prime editors, which include the use of dual AAVs, or non-viral vectors (e.g., liposomes), which additionally can be used for delivering Cas protein instead of DNA.

The eye and ear are particularly suitable organs for a wide range of therapeutic interventions because: 1) there is a minimal risk of therapeutic agents diffusing to unwanted tissues or organs upon local delivery, due to the confined environment of the compartments concerned, 2) these organs have a fluidic composition, favoring dissemination to target cell; and 3) the two organs are unique zones of immune privilege, thanks to a blood-organ barriers impeding movement of cells and molecules into and out of the sensory epithelia (Bucher et al., 2020). Over the years, different vectors and delivery routes have been tested, and their efficiency validated in the two sense organs (Delmaghani and El-Amraoui, 2020; Sahu et al., 2021; Nyberg et al., 2019; Yu and Wu, 2021; Valentini et al., 2020; Wang et al., 2019; see also Figure 4). Details on viral and non-viral delivery vehicles, routes of administration approaches into the eye and the inner ear can be found in recent reviews (Nyberg et al., 2019; Wang et al., 2019; Delmaghani and El-Amraoui, 2020; Valentini et al., 2020; An and Zha, 2020; Sahu et al., 2021; Yu and Wu, 2021; Toualbi et al., 2021).

As mentioned above, AAV vectors are currently the leading choice for *in vivo* gene transfer studies, with over 40 gene therapy clinical trials, thanks to their non-pathogenicity, low immunogenicity and their ability to mediate persistent transgene expression. Numerous AAV serotypes and variants have been tested using different injection routes into the eye and the inner ear, documenting their safety and efficiency to target specific retinal or inner ear cell types (Buck and Wijnholds, 2020; Fakhiri et al., 2020; Lan et al., 2020). The capsid serotype and promoter in front of the transgene are key parameters determining expression in a given cell type. Novel AAV capsids, and cis-regulatory elements (such as short promoters, enhancers, polyadenylation sequences) are being continuously improved to further expand the utility of AAV mediated gene therapy in ocular and auditory disorders (Planul and Dalkara, 2017; Sahu et al., 2021). One note-worthy method for improving capsid proteins has been directed evolution generating optimized AAV capsids for specific gene therapy applications (Zinn et al., 2015; Byrne et al., 2020). Among the new AAVs, AAV2-7m8, AAV2/8BP2, NHP9, NHP26, AAV2.GL were shown to display an improved capacity to transduce retinal photoreceptor cells (Cronin et al., 2014; Byrne et al., 2020; Garita-Hernandez et al., 2020; Pavlou et al., 2021) and some of these have also the capacity to transduce efficiently the auditory hair cells (Landegger et al., 2017; Isgrig et al., 2019). Some variants for improved neural gene delivery after systemic administration have also been used to target auditory hair cells (e.g., AAV9-PHP.B, (György et al., 2019a; Wu et al., 2021). Other interesting methods such as rational design (Petrs-Silva et al., 2011), in silico ancestral design (Zinn et al., 2015; Landegger et al., 2017) and chemical modification of AAV capsids (Mével et al., 2019) have also provided variants valuable for gene delivery to the inner ear and retina. These efforts in capsid design are complemented by the discovery and use of new promoters specific for unique or subpopulation of retinal cell types (Jüttner et al., 2019).

The constant development of new generations of AAVs will expand further the scope of the target cells and organs for AAVs transduction, but the major limitation to their applications will remain their relatively small packaging capacity (Trapani et al., 2020). Only a DNA fragment of no more than 5 kb, including the two 145 bp ITRs, can be efficiently packaged into an AAV vector. A large number of human disease genes and the great majority of gene editing machineries [e.g., the commonly used *Streptococcus pyogenes* Cas9 (SpCas9), the base and prime editors, along with related sgRNAs] are too large to fit into a single AAV. To overcome this size limitation, oversized AAV vectors and dual AAVs have been tested, with significant improvements of retinal or hearing features in defective animal models (Omichi et al., 2020; Trapani et al., 2020; Maddalena et al., 2018; Reisinger, 2019; Akil, 2020). The first test of dual AAV vector approach in humans is underway in the eye, to monitor the safety and efficacy of dual AAVs expressing the Usher syndrome type 1B gene, MYO7A (https://www.ushther.eu/). Dual AAVs has also been successfully used to deliver gene editors *in vivo* (Moreno et al., 2018). Several newly discovered Cas enzymes have shorter coding sequences, which allows the packaging of both Cas and sgRNA into a single AAV vector leading to more efficient genome editing. These include the *Staphylococcus aureus* Cas9 (SaCas9) (Ran et al., 2015), the *Campylobacter jejuni* (CjCas9) (Kim et al., 2017a), and other variants such as St1Cas9, NmCas9, SaCas9-KKH, CasX, Cas12 (see Anzalone et al., 2020; Saha et al., 2021).

In parallel, other viral vectors with large packaging capacity have been used over the years, some were even used in phase I/II clinical trials. Adenoviral and Lentiviral vectors have larger capacity (∼8–10 kb) and can stably transduce both dividing and non-dividing cells *in vivo*. The adenoviral vectors were the first ocular gene delivery tools used in mice, and vector-mediated reporter gene expression was detected in RPE and photoreceptor cells (Bennett et al., 1996; Yu and Wu, 2021). Novel adenoviruses have later been developed to reduce their immunogenic features (Husseman and Raphael, 2009; Shu et al., 2016), some being used in clinical trial after successful outcomes in animal models (Math1, NCT02132130). Similarly, 3rd generation lentivirus such as Equine infectious anemia virus (EIAV) have being used in the eye to express the MYO7A gene (6,645 bp; 2,215 amino acid) and the ABCA4 gene (6,819 bp; 2,273 amino acids) in clinical trials for Usher syndrome type IB and Stargardt disease (NCT01367444; NCT01505062), respectively. Lentiviral vectors efficiently infect RPE but it is less clear how these vectors can mediate efficient transduction of photoreceptor cells (Puppo et al., 2014). To foster progress in future applications of such vectors, it is of utmost importance that the findings of the completed and ongoing related-clinical trials are made public, to enable informed decision on follow-ups application for adenoviral- and lentiviral-based approaches.

Compared to viral-mediated transfer, non-viral delivery approaches have the advantage to deliver the therapeutic agents in the form of protein or mRNA, which avoids the possibility of permanent recombination into the genome and limits the risks of genotoxicity and unwanted off-target effects. Cationic lipids have been shown to form complexes with Cas9 or the new editing agents and their gRNAs, facilitating correct transfer into the targeted cells (Zuris et al., 2015). Devolvere et al. were the first to demonstrate, *in vivo,* the capacity of cationic lipids to deliver mRNA to the photoreceptor layer, leading to production of the corresponding protein 7 days after subretinal injection in mice (Devoldere et al., 2019). In a second study, high levels of protein were reported in the RPE following the delivery of mRNA to the retina with an ionizable lipid (Patel et al., 2019). This non-viral strategy was first used *in vitro* and in the inner ear by the group of David Liu in 2014. It was first applied to the eye by Kim et al., 2017, who showed that cationic non-viral vectors were able to deliver Cas9 protein to the RPE, resulting in indels in 22% of the transfected cells. Transient expression was ensured by degradation of the Cas9 protein 3 days after subretinal injection (Kim et al., 2017b). Holmgaard et al. confirmed that it was feasible to transfer the Cas9 protein directly to the RPE with lipid vectors, to disrupt the VEGFa gene. They achieved an indel rate of 6% in the VEGFa gene in isolated EGFP-positive RPE cells (Holmgaard et al., 2021). However, they reported signs of toxicity at high Cas9/gRNA concentrations (Holmgaard et al., 2021). This approach has also been shown to be effective in mouse inner ear cells (Zuris et al., 2015) and in a mouse model of genetic deafness, in which it led to phenotypic improvement (Gao et al., 2018). Recently, Chen et al. induced efficient genome editing in the RPE by conjugating their nanoparticles with all-trans retinoic acid which are selectively transported to RPE by the inter-photoreceptor retinoid-binding protein (Chen et al., 2019). However, potential of non-viral vector to deliver Cas9 to the retina has not yet been reported.

Liposome-based delivery has been shown to be effective for targeting inner ear cells at neonatal stages, but its efficacy is lower in the fully mature organ (Zuris et al., 2015). Further studies are required to determine whether effective delivery vehicles for gene editing can be used to target specific retinal and inner ear cell types, and lead to efficient gene editing (Li et al., 2017; Mittal et al., 2019; Anzalone et al., 2020; Saha et al., 2021). Nonetheless, current exiting editors (cf. below) offer opportunities to test and evaluate efficacy of a wide range of treatment possibilities to restore hearing and vison using available animal models. In the sections below, we will focus on CRISPR-Cas based technologies and their application *in vivo* in the eye and inner ear.

## Gene Editing for Eye and Inner Ear Gene Therapy

Clinical trials of gene editing tools are already underway for several specific human conditions, opening new possibilities for the use of novel therapies for human genetic and/or epigenetic disorders based on gene and RNA editing (Anzalone et al., 2020).

**FIGURE 5:**
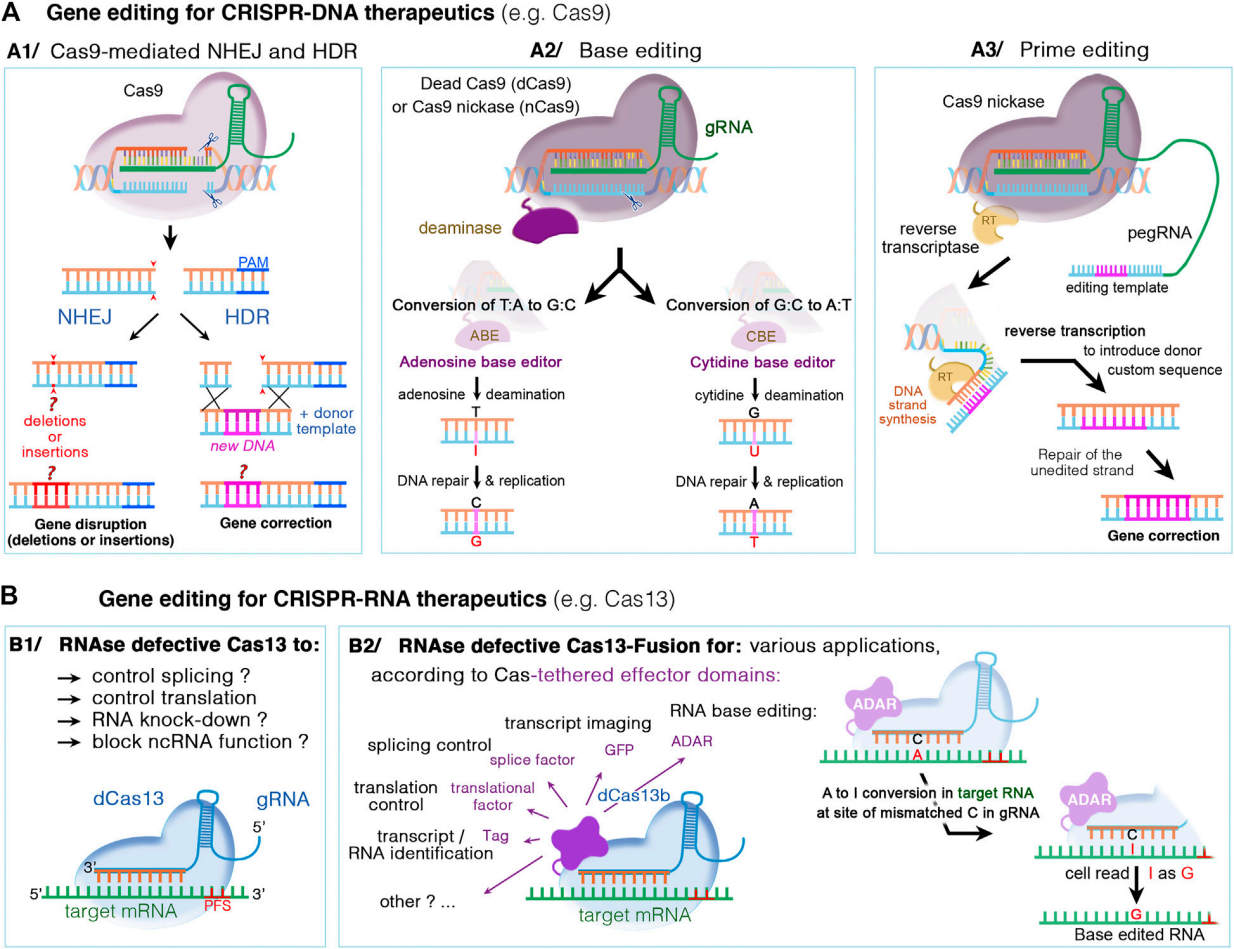
DNA- and RNA-based genome editing to manipulate disease gene-of-interest. Different mechanisms occur, depending on the type of CRISPR/Cas nuclease and supplied editing tools. **(A1)** The DNA CRISPR/Cas nucleases such as Cas9 or Cas12 can bind and cleave the genomic DNA. The double-stranded DNA breaks can then undergo repair by either the nonhomologous end joining (NHEJ) or homology-directed repair (HDR). NHEJ is a random and highly error prone mechanism that incorporates insertion/deletion mutations, called indels, causing gene disruption (or silencing). In HDR, the DSB can be repaired by externally adding a donor DNA template that is homologous to the target sequence. The donor template is copied into the targeted site, resulting in a directed precise repair of the defective DNA sequence. **(A2)** Base editing relies on inactivated Cas nucleases (dcas) with preserved DNA targeting but without DNA cleavage, and could thus be used in non-dividing cells. The dCas nuclease (for instance Cas9 nickase, nCas9) is coupled to a deaminase, cytidine (CBE) or adenosine (ABE), which make it possible to edit specific nucleotide without DNA double break: C-to-T transitions by CBE editors or A-to-G transitions by ABE editors. **(A3)** Prime editing is suitable to edit both point mutations and larger deletions or insertions. Here, dCas9 is fused with an engineered reverse transcriptase enzyme (RT), combined with a prime editing guide RNA (pegRNA) that serve to both position the enzyme at target site and provide the template sequence necessary to correct or replace the defective DNA region. **(B)** CRISPR‐Cas RNA systems, such as Cas13, can be used to manipulate cellular RNA both for basic research and therapeutics. While catalytically active Cas13 variants can cleave and disrupt the targeted RNA, the RNase-defective dCas13 **(B1)** and dCas13-effector fusion **(B2)** variants further expand possible RNA manipulations. These include regulation of RNA stability, splicing, intracellular localization, epitranscriptome modulation, translational activation/repression, RNA imaging, labeling of RNA‐interacting proteins, or site-directed nucleobase editing (possible by ADAR effector domains), Abbreviations: ADAR, adenosine deaminase acting on RNA; PFS, protospacer flanking sequences; A, adenosine; T, thymidine; C, cytidine; G, guanosine; I, inosine; and U, uracile.

Over the last years, class II CRISPR systems such as Cas9 and Cas12 have been widely used for programmable RNA-guided DNA targeting. The CRISPR-Cas system consists of two key molecules: an enzyme called Cas, and a guide RNA (gRNA) located within a longer RNA scaffold. The scaffold RNA binds to the Cas protein and the predesigned sequence “guides” the enzyme to the part of the genome targeted for modification (see Figure 4). The gRNA is designed to identify and bind to a specific sequence in the DNA. The Cas follows the gRNA to the target site in the DNA sequence, at which it cuts both strands of the DNA right upstream of the protospacer adjacent motif (PAM) in the genome (Figure 5A1). Unlike the PAM-proximal blunt-end cuts generated by Cas9, Cas12 nucleases typically generate staggered cuts within regions of the protospacer that are distal to the PAM sequence, which is advantageous for applications such as integrating DNA fragments in a preferred orientation (Anzalone et al., 2020). To increase editing specificity and to widen the scope of sequence recognition through various PAM motifs, dozens of Cas orthologs were discovered and engineered (e.g., SmacCas9, SaCas9, SaCas9-KKH, FnCas9-RHA, enAsCas12a, LbCas12a and AsCas12a, …; see Anzalone et al., 2020; Saha et al., 2021; Yu and Wu, 2021 for a more exhaustive list). The damaged DNA undergoes repair, predominantly by mechanisms naturally present in mammalian cells: mainly, the non-homologous end joining repair (NHEJ) resulting in insertions and deletions (referred to as indels) (see Figure 5A1). In the presence of a donor DNA template (e.g., single-stranded oligonucleotide donors ssODNs), a homology-directed repair (HDR) mechanism can occur, mostly in dividing cells. This competing (typically less efficient) pathway is used to correct targeted mutations or to knock in larger DNA sequences. However, the efficiency of HDR following CRISPR-Cas-mediated double-strand DNA breaks (DSBs) *in vivo* remains low. This challenge has fostered searches for other strategies for improving DNA repair (Figures 5A2–3) (Levy et al., 2020).

A new generation of gene editing systems recently emerged, broadening the scope of targetable sequences in the genome, and expanding their use beyond gene disruption and exon skipping (Anzalone et al., 2020). These new developments include the base and prime editors, which offer the potential for the effective and permanent correction of pathogenic mutations *in vivo*, in post-mitotic cells, without causing DSB and with the advantage of minimizing the formation of indels or off-target mutations (Levy et al., 2020; Niggemann et al., 2020). The DNA base editors developed by the group of David Liu induce the deamination of either cytosines or adenines, to allow for the repair of transition mutations (C-T or A-G changes) (Huang et al., 2021). Cytosine base editors (CBE) can convert C:G nucleotide pairs into A:T nucleotides (Komor et al., 2016), and adenine base editors (ABE) convert A:T into C:G nucleotides (Gaudelli et al., 2017). Prime editor (PE) has further expanded the CRISPR base-editing toolkit to all 12 possible transition and transversion mutations, and to small insertions or deletions (Anzalone et al., 2019) (Figure 5A3). This new process involves the use of an inactive Cas fused to a reverse transcriptase and an RNA called the pegRNA, which contains both the guide RNA and the correct template sequence (see Figure 5A3). Interestingly, prime editors are able to install point mutations at distances far (>30 bp) from the site of Cas9 nicking, which offers greater targeting flexibility than nuclease-mediated HDR with ssDNA donor templates, which typically are unable to introduce edits efficiently more than ∼10 bp from the cut site (Anzalone et al., 2020). Finally, the recent progress in gene editing also involves new site-directed editing systems to edit RNA rather than DNA. This offers an improved safety profile, due to the transient and potentially reversible nature of edits made to RNA (Rees and Liu, 2018; Fry et al., 2020, see also Figure 5B). Current progress in RNA editing technologies enabled the development of engineered enzymes capable of either adenosine-to-inosine (A-I, see Figure 5B2) or cytosine-to-uracil (C-U) edits (Fry et al., 2020). Besides site-directed nucleobase editing, the use of inactive RNA enzyme editors tethered to various effector domains expand their applications to include regulation of RNA stability, splicing, intracellular localization, epitranscriptome modulation, translational activation/repression, RNA imaging, labeling of RNA‐interacting proteins (see Figure 5B2).

## Gene Editing in Inherited Blindness and Deafness

The advent of CRISPR-based approaches has opened a new avenue for gene therapy development in rare diseases. As described above, few hundred genes whose defect leads to isolated and/or syndromic vision (RetNet: https://sph.uth.edu/RetNet/) or hearing (http://hereditaryhearingloss.org/) loss have been reported. Commonly sought edits in these diseases include the deletion or insertion of DNA base pairs, conversion of DNA base pairs, or a combination of these changes. The most appropriate gene editing strategy (silencing and/or repair) depends on the form of deafness treated and its mode of transmission. The type of editing required determines the choice of nuclease or editor (+/− epigenetic modifier), the availability of appropriate protospacer adjacent motif (PAM) sites, and the dynamic range of the therapeutic effect. According to ClinVar (ncbi.nlm.nih.gov/clinvar), ∼82–84% variants associated with inherited retinal dystrophies or nonsyndromic deafness are substitutions (Landrum et al., 2016; Niggemann et al., 2020). The CBE base editors can correct ∼34 and 10% of A-G changes causing vision or hearing loss, respectively, while ABEs can correct ∼28 and 37% causing vision or hearing loss, respectively. Additional mutations such as transversions (26% in deafness, ∼22% IRDs), small indels (26% in deafness, 15% IRDs) and duplications (3% IRDs), could potentially be repaired by prime editors. In theory, the genome-editing techniques currently available can be used to treat up to 99.9% of genetic defects associated with hearing and vision loss. Many attempts have been made in recent years to demonstrate the efficacy of gene-editing tools for correcting human mutations, in cultured cell lines, and therapeutically relevant iPSCs with blindness- and/or deafness-associated mutations (Sanjurjo-Soriano et al., 2020; Nourbakhsh et al., 2021; Stojkovic et al., 2021; Zine et al., 2021). We focus here on the principal promising data obtained *in vivo* for retina- and inner ear-related disorders. These include gene inactivation/repression for dominant or gain-of-function mutations, as well as attempts for precise correction of the causal mutations.

### Gene Silencing of Blindness or Deafness Causal Mutations in the Eye and the Ear

The current programmable nucleases have proven to be more effective for gene knockout or for the excision of specific regions of genomic DNA. Gene knock-out and deletion using the NHEJ repair pathway has thus been tested for the treatment of hereditary diseases with dominant or gain-of-function gene mutations.

### Gene Silencing in Dominant Forms of Inherited Retinal Disorders

Gene inactivation by a Cas9/gRNA DSB approach is useful for dominant genetic defects, provided that the mutated allele is targeted specifically. Two strategies for the successful application of this gene inactivation *in vivo* in the retina have been developed, the first is based on an allele-specific PAM sequence present only in the mutated allele (Giannelli et al., 2018; Li et al., 2018), and the second is based on a mutation-specific gRNA (Bakondi et al., 2016). Representing 25% of dominant retinitis pigmentosa and 15% of all the retinal degenerations, the RHO gene encoding rhodopsin is among most frequently studied genes (Dryja et al., 1990; Sung et al., 1991). Among all the mutations in the RHO gene, P23H mutation is the most common one in North America leading to misfolding and aggregation of the protein causing a dominant-negative effect in rod photoreceptors. Two studies took advantage of the engineered variant Cas9-VQR or its improved version Cas9-VRQR that recognize the novel PAM NGA to precisely target the P23H mutated allele in knock-in P23H mice (Giannelli et al., 2018; Li et al., 2018). Giannelli et al. delivered by an intravitreal injection two AAV-PHP.B vectors, encoded SaCas9-VQR under photoreceptor specific promotor and sgRNA with a GFP reporter (Giannelli et al., 2018). They demonstrated a selective inactivation of P23H allele and an efficiency up to 27% indels among the highly GFP positive sorted cells (Giannelli et al., 2018; Li et al., 2018). In a second study, Li et al. found higher editing efficiency with the improved version of Cas9-VRQR compared to Cas9-VQR. The majority of edited cells display a knockout of P23H mutated allele and a subsequent decrease of its transcripts (Giannelli et al., 2018; Li et al., 2018). To ensure an allele-specific inactivation, Bakondi et al. based their strategy by two base pair differences between the mutated and WT allele, one inside the PAM and one at position 10 in the 20 pair bases gRNA. This system allowed the discrimination of the two alleles in a transgenic S344ter rat, a model of severe dominant retinitis pigmentosa characterized by rapid and progressive loss of outer nuclear layer from P11 to P28 (Bakondi et al., 2016). These three studies showed the potential of specific allele disruption to preserve rod photoreceptors in dominant retinal diseases. However, these mutation-dependent strategies render treatments expensive and are beneficial to only a small number of patients. Moreover, it is not possible to compensate for haploinsufficiency due to inactivation of the mutant allele, and discrimination between the wild-type and mutated alleles is not always possible.

In this context, silencing and replacement strategies have been developed for dominant disease. The approach used involves the non-specific disruption of the two alleles, followed by their replacement via an AAV-based method. Latella et al. reported the inactivation of the RHO gene in neonatal P23H RHO transgenic mice after subretinal injection followed by electroporation of three plasmids encoding Cas9 DNA, two sgRNAs flanking exon1 and a GFP reporter. Among the GFP positive cells, a significant decrease in RHO protein levels was observed (Latella et al., 2016). In addition to the silencing of mouse RHO gene by Cas9 and two sgRNAs, Tsai et al. used a dual AAV approach to deliver a human RHO cDNA in postnatal P23H and D190N RHO knock-in mutant mice. Interestingly, they split the Cas9 and the sgRNAs into two AAVs and observed a correlation between the decrease of mRHO and the increase of hRHO levels. After 3 months, a phenotypic improvement was observed in the two models suggesting the possibility to apply this strategy in a mutation-independent way. In a third study, McCullough et al. provided proof-of-concept for the gene disruption part of the strategy in non-human primate in the context of the autosomal dominant cone-rod dystrophy (CORD6). CORD6 is mainly caused by mutations in GUCY2D gene coding for the retinal guanylate cyclase 1 (retGC1) (McCullough et al., 2019). Dual AAVs were used to encode saCas9 under photoreceptor-specific promoter GRK1 promoter and a gRNA targeting GUCY2D gene in fusion with a GFP reporter. Among sorted GFP positive photoreceptors, up to 13% editing efficiency (indels and AAV vector insertions) was observed, leading to 80% of retGC1 disruption. However, the second part of the strategy, involving the simultaneous replacement of the endogenous gene by a healthy gene, has yet to be reported and remains challenging (McCullough et al., 2019).

### Gene Silencing in Dominant Forms of Hearing Disorders

The groundwork for gene editing in the inner ear *in vivo* was performed on the transgenic Atoh1-GFP mouse cochlea, in which all hair cells express GFP under the control of a hair cell-specific enhancer for the transcription factor Atoh1 (Zuris et al., 2015). In this work, 2 weeks after neonatal lipid-mediated transfer of Cas9 and its gRNA targeting GFP-Atoh1, GFP expression was disrupted in about 20–25% of the outer hair cells (Zuris et al., 2015). This *in vivo* targeted gene disruption clearly suggests that mutations can be efficiently disrupted in hair cells, potentially leading to hearing recovery. Studies in animal models of deafness have been initiated, to confirm the efficacy of gene editing in the auditory hair cells. Given the high rate of gene editing mediated NHEJ, “switching off” the expression of the mutated allele while leaving the non-mutated DNA untouched may be an appropriate approach for dominant inherited forms of deafness. In this context, Gao et al. were the first to develop a gene-editing approach targeting DFNA36, a non-syndromic form of deafness with a dominant mode of inheritance (Gao et al., 2018). The mutated gene, *TMC1* (transmembrane channel-like 1), is expressed in hair cells and encodes a subunit of the mechanoelectrical transduction channel. Interestingly, in the Beethoven (*Bth*) mouse model of deafness, hearing loss is caused by a missense *Tmc1* mutation (p.M412K, c.T1235A) and follows a dominant mode of inheritance. This mutation is orthologous to the human *TMC1* mutation, c.1253T > A (p.M418K), found to be responsible for DFNA36 in a Chinese family (Zhao et al., 2014). Untreated *Tmc1* ^Bth/+^ mice display a progressive increase in auditory response threshold and progressive hair cell loss, beginning at the age of 1 month (Vreugde et al., 2002). The cationic lipid-mediated Cas9–single guide RNA complex was targeted to the mutant Tmc1 allele by direct injections into the scala media via cochleostomy in neonatal *Tmc1* ^Bth/+^ mice. A slight restoration was observed in the treated ears, even 8 weeks post-treatment, but the observed improvement was limited to frequencies between 8 and 23 kHz (with mean ABR thresholds 15 dB lower for treated ears than for the untreated contralateral ears). The significant but modest degree of hearing preservation (less than 20 dB) observed is consistent with the small number of hair cells corrected, and, perhaps, with a lack of specificity of Cas9 for the mutant allele. Indeed, analyses of the sequences of 12,000 reads containing indels revealed that 6% contained modifications of the wild-type *Tmc1* allele. One recent study evaluated the selectivity of different Cas9 and gRNA combinations as a means to improve specificity for the mutant allele (*Bth*, c.1253A), which differs from wild-type *Tmc1* by only one base pair (György et al., 2019b). In studies using the *Streptococcus pyogenes* Cas9 (SpCas9) in combination with 12 different full-length and truncated gRNAs targeting *Tmc1*exposant or *Tmc1*exposant, indel events clearly occurred in both the *Tmc1*exposant and *Tmc1*exposant alleles. These findings indicate that SpCas9 can tolerate mismatches between gRNAs and can be used to target the *Bth* allele. By contrast, use of the *Staphylococcus aureus* Cas9 (SaCas9-KKH) with a PAM specifically selected to recognize the mutated residue, resulted in a more efficient effect that was selective for the mutant (c.T1253A), but not the c.T1253 wild-type *Tmc1/TMC1*. Efficiency and selectivity for the mutated allele were first confirmed in a DFNA36 human cell line harboring the c.1253T > A (p.M418K) mutation. Further studies *in vivo* showed that AAV (Anc80L65)-mediated delivery of the SaCas9-KKH-gRNA complex efficiently prevented deafness in Bth mice for up to 1 year post injection (György et al., 2019b). The DPOAE thresholds of the treated Tmc1Bth mice revealed a preservation of OHC function at lower frequencies (5–11 kHz) at 12 weeks of age, and in animals surviving until 24 weeks of age. In treated mice, the hair bundles of cochlear OHCs and IHCs (in the 8 and 16 kHz regions) and vestibular hair cells recovered normal morphological features after treatment, with minimal hair cell loss. Similar PAM-selective strategies for allele-specific disruption with SaCas9-KKH could be used for other dominant disease mutations. An analysis of the sequences of dominant disease variants reported in the ClinVar database identified about 3,759 pathogenic variants as suitable for targeting by a similar approach. This approach has been recently used successfully to correct a semi-dominant mutation in the myosin VI gene, *Myo6*, using a mouse mutant the reproduce the human DFNA22 deafness mutation (Vaerman and Heremans, 1968).

### Gene Silencing in Recessive Forms of Sensory Disorders

This has proved to be the most easily applied strategy for the delivery of SaCas9 and its gRNAs with a single AAV vector to the retina *in vivo*. The most advanced applications to date have involved the use of this technique to correct recessive Leber congenital amaurosis type 10 (LCA10), which is caused by an intronic mutation of the CEP290 gene. The intronic c.2991+1655A > G mutation, reported in 20–57% of LCA10 patients of European descent, generates a new splice donor site leading to the insertion of a cryptic exon (exon X) in CEP290 mRNA (p.Cys998X), leading to a premature stop codon in 50% of *CEP290* transcripts (Perrault et al., 2007). A combination of two gRNAs and a SaCas9 which is under the photoreceptor-specific promoter GRK1 is delivered subretinally with a single AAV5, for direct excision of the intronic mutation. This method was shown to be safe and feasible in non-human primates with a dose dependent response and an efficiency superior to 10% productive edits, minimum threshold determined as clinically efficacious (Maeder et al., 2019). This study leads to the first clinical trial based on gene editing in the eye (NCT03872479).

### Gene Silencing to Trigger Pathway-Induced Cell Reprogramming in the Eye and the Ear

It can be costly to correct each pathogenic variant individually. The development of gene-independent strategies based on CRISPR-Cas9 tools expand their application to non-inherited forms of the sensory disorders increasing treatment accessibility. CRISPR-based gene-editing tools can be used for other purposes in mammalian cells, including the activation or repression of genes of interest, and the epigenetic reprogramming of cellular identities.

In the retina, two groups have investigated the feasibility of a broadly applicable treatment for rod-cone dystrophies that is independent of the causal gene. This approach is based on repression of the Nrl gene, which can lead to the *in situ* reprogramming of rods cells into cone-like cells resistant to RP-specific mutations (Yu et al., 2017; Moreno et al., 2018). Genome editing is also a potentially powerful tool for the treatment of non-genetic degenerative diseases. Two studies reported an *in vivo* genome-editing approach for treating age-related macular degeneration (AMD) (Kim et al., 2017b; Holmgaard et al., 2021). Using cationic lipids vectors, they showed that Cas9/gRNA approaches based on disruption of the VEGFa gene could have therapeutic effects *in vivo* in a mouse model of AMD (Kim et al., 2017b).

In the inner ear, substantial evidence suggests that oxidative stress-induced apoptosis and necrosis in cochlear cell types, including hair cells, underlie drug-induced inner ear damage leading to hearing loss (Leis et al., 2015). A recent study harnessed CRISPR/Cas9 editing agents to tackle this apoptotic pathway by targeting the *Htra2* gene, which encodes a proapoptotic mitochondrial serine protease. Because *Htra2* was identified among a set of highly overexpressed genes after drug-induced inner ear damage, two CRISPR/Cas9 systems, SpCas9 and SaCas9, were used to disrupt its expression in the inner ear (Gu et al., 2021). The CRISPR/Cas9-mediated inhibition of Htra2 significantly decreased neomycin-induced apoptosis, promoted hair cell survival, and improved hearing function in drug-treated mice. The best results were obtained using the Anc80L65–SaCas9–Htra2 gRNA system, probably thanks to a more efficient transduction rate of the hair cells using single AAVs (Gu et al., 2021). The injected ears showed sustained (up to 8 weeks) and significant improvement in auditory brainstem response threshold, up to 50 dB at 8 kHz for the SaCas9 system (Gu et al., 2021). The protective effect did not cover all sound frequencies, as no beneficial outcomes were observed in the basal turn of the cochlea. Besides the improvement of editing efficiency for better outcomes, safety issues need be considered to document potential side effect of a permanent gene inhibition, within and beyond the inner ear.

### Precise Correction of Blindness and Deafness Causal Mutations

Precise correction has clear advantages over silence-and-replace strategies and would be applicable to diseases due to both dominant and recessive defects of large genes. However, the NHEJ repair pathway predominates in post-mitotic retinal cells, and repair via the HDR pathway would therefore be unlikely to lead to a satisfactory correction rate *in vivo*.

The first strategy that could be used to overcome this limitation is the homology-independent targeted integration (HITI) strategy, which bypasses the HDR pathway and uses the predominant NHEJ pathway to accurately integrate sequences of interest at a given site (Suzuki and Izpisua Belmonte, 2018). In this approach, the Cas9/gRNA complex targets both the genome sequence and two short sequences inserted on either side of the donor template. Suzuki et al. provided proof-of-concept *in vivo,* in a rat model of retinitis pigmentosa. They used a dual AAV approach for the successful correction of a homozygous mutation of the Mertk gene in RP, leading to partial rescue of visual function (Suzuki et al., 2016). However, a mosaic of editing products can be generated by HITI strategy, as indels without HITI are still created by the NHEJ repair pathway.

Two studies have already reported the use of base-editing strategies in the retina of mice (Levy et al., 2020; Suh et al., 2021). In the first study, Levy et al. reported the use of a split-intein base editor-dual AAV strategy in mice expressing tdTomato only in rod photoreceptor cells (Levy et al., 2020). Robust base editing was reported in transduced rod photoreceptors suggesting that this delivery system could achieved therapeutic editing efficiencies *in vivo*. In the second study, a lentivirus expressing the ABE protein and its gRNA was delivered to the RPE of LCA mice model by subretinal injection, leading to ∼16% of correction of a *de novo* nonsense mutation in the RPE65 gene and the restoration of Rpe65 expression (Suh et al., 2021). To assess the rescue of visual function, retinal cell activity was measured using scotopic electroretinography (ERG), the recovery of 44 and 65% of a- and b-wave amplitudes were observed compared to WT control responses.

In the inner ear, cationic lipid-mediated (Gao et al., 2018) and AAV/Anc80L65-mediated (György et al., 2019b) delivery of Cas9/gRNA complexes have been successfully used to edit mutations in TMC1 gene, responsible for deafness. These studies resulted in satisfactory levels of specificity and efficacy in the middle and apical regions of the cochlea, but no restoration of hearing and cellular architecture was observed in the region responsible for processing high-frequency sounds (32 kHz). This result probably reflects inefficient transduction of the auditory hair cells at the base of the cochlea, which continued to degenerate, as in untreated mice. Additional investigations should be performed, with better delivery methods and agents. Regarding TMC1 gene, at least 59 mutations (of the 67 reported in ClinVar) are recessive deafness-causing mutations. The ability to correct recessive loss-of-function mutations would therefore have broad clinical implications. Yeh et al. (2020) evaluated base editing for the correction of a deafness mutation in the *Baringo Tmc1*exposant mouse model, which carries a recessive point mutation in Tmc1, c.A545G, resulting in profound hearing loss by the age of 4 weeks (Manji et al., 2012). However, as mentioned above base editors (∼5.2 kb) are too large to fit into a single AAV. Alternative delivery approaches are therefore required, such as the split-intein, dual-AAV base editor delivery system, in which a cytosine base editor (CBE) is split into two halves, each of which is delivered by a separate AAV. Prior tests of combinations of CBE and guide RNA were performed *in vitro*, in mouse embryonic fibroblasts from *Baringo* embryos, with several CBEs and gRNAs packaged into a split-intein, dual-AAV. The ability of each combination to correct the pathogenic *Tmc1*exposant C•G base pair directly to the wild-type T•A base pair was assessed. Two weeks after injection, the efficiency of base editing in the inner ear ranged from 10 to 51%. In the treated ears of Baringo mice, hair bundle morphology and mechanoelectrical transduction activity were restored in 64–75% of the inner hair cells (notably those in the apex of the cochlea). ABR recordings revealed a 5–50 dB improvement in auditory function in nine treated mice, with ABRs remaining undetectable in untreated mice. However, DPOAE measurements revealed no recovery of OHC activity, probably due to the lower viral transduction efficiency for Anc80 in OHCs: 2.6–8.3% of OHCs were transduced, versus 23–42% of IHCs (Yeh et al., 2020). Wu et al. recently made use of the high rate of auditory hair cell transduction with AAV9-PHP.B vectors to use base editing to correct the *Tmc1 Baringo* mutation (Wu et al., 2021). The AAV9-PHP.B dual AAVs were able to transduce OHCs, leading to a better restoration of DPOAE thresholds in treated mice, reflecting a stronger recovery of OHC function. DPOAE thresholds were even lower than those of WT mice after treatment in some conditions, raising the interesting possibility that Tmc1 overexpression might lead to an increase in protein stability or turnover, thereby enhancing OHC function. Further studies with higher rates of target cell transduction are required to demonstrate the specificity and efficacy of treatment at later stages.

### Base Editing to Trigger Pathway-Induced Cell Reprogramming in the Inner Ear

Base editors can be used not only to edit genetic mutations, but also to alter various biological processes. The β-catenin pathway has been implicated in hair cell regeneration through the activation of Atoh1, a transcription factor responsible for determining hair cell fate (Shi et al., 2010). Thus, treatments activating the β-catenin pathway are being explored as a means of stimulating hair cell regeneration. With CBE base editing, the codon TCT encoding Ser33 (a phosphorylation site) was converted to TTT, creating β-catenin S33F. This modification blocks β-catenin phosphorylation, impedes β-catenin degradation by blocking protein ubiquitination, and upregulates Wnt signaling. In neonatal mice, this codon conversion was performed by Lipofectamine 2000-mediated RNP delivery, and led to the promotion of cell division and trans-differentiation into hair cells (Yeh et al., 2018). The resulting Wnt activation induces the mitosis of cochlear supporting cells, and cellular reprogramming to produce hair cells. These data further expand the applications of gene editing to the modification of posttranslational states in signaling pathways as a means of preventing or correcting degradation of the sense strand.

## Conclusion and Prospects for Applications of Gene Editing *In Vivo*

Gene editing therapies are rapidly progressing, and we expect several attempts will soon reach the clinical trial stage. Currently registered clinical trials using editing agents are dominated by *ex vivo* modifications, in which the cells are altered in the laboratory, carefully checked and assessed for safety and then transferred back to the patient. In addition to these *ex vivo* trials (ongoing for HIV, cancer, and blood disorders), there have been promising advances in treatment *in vivo* for readily accessible tissues, such as the cervix, liver (NCT03041324 and NCT04601051), eye NCT03872479) and ear. The sensory organs of the eye and inner ear will undoubtedly continue to drive progress in genome editing *in vivo*, because they are easy to access and self-contained, minimizing systemic effects.

From a therapeutic perspective, expectations in term of restoration outcomes in the inner ear and the retina are different. Correct targeting the auditory hair cells all along the spiral cochlea, from the base to the apex of the hearing organ, is key to normal hearing and speech discrimination. Indeed, the restoration of cochlear tonotopy and the related ability to perceive sounds at high, medium and low frequencies is dependent on the correction of as many hair cells as possible along the cochlear partition (Delmaghani and El-Amraoui, 2020). By contrast, it may not be necessary to target all the retinal photoreceptors to restore some useful vision. In Humans, correction of a fraction of the 120 million photoreceptor cells, in a given region of the retina such as the cone-rich fovea, is likely sufficient for the restoration of useful levels of vision. Indeed, it is believed that 50% of cone function in the fovea is compatible with 20/20 vision, and 95% cone loss is compatible with correct orientation and discrimination performance (Geller et al., 1992; Geller and Sieving, 1993). Also, subretinal injections covering parts of the retina have been sufficient to restore enough vision for the treated patients to perform tasks important to the patient quality of life (Russell et al., 2017; Cehajic-Kapetanovic et al., 2020).

Thanks to ongoing promising success of gene editing therapies in the eye and the inner ear, increasing proof of concept studies in these organs are expected in the near future. However, while the CRISPR nucleases, and the new generations of base, prime, and RNA editors bring highly versatile new tools to precision genome editing, each of these approaches comes with its own advantages and limitations. Increased efforts by researchers worldwide, from multidisciplinary fields, are put together to accelerate the development of gene editing solutions that are both highly efficient and safe, without toxicity and minimal activation of innate immune responses is pushing this aim further. In this context, the (NIH) Somatic Cell Genome Editing consortium (38 institutions) established a roadmap of efforts to develop and benchmark approaches to induce and measure genome modifications, and to define downstream functional consequences of genome editing within human cells (Saha et al., 2021). Besides safety issues, sought goals include the improvement of the editing efficiency (the fraction of the intended loci that are edited), the precision [the relative frequency of desired (for example, reversion of a pathogenic allele) versus undesired (for example, large deletions or translocations) modifications at the intended loci] and the accuracy (how many off-target sites are unintentionally edited, and to what extent) of gene editing agents (Saha et al., 2021). Further studies are required to identify the best delivery method and gene editing design for preventing innate immune responses to components of the gene editing machinery. Indeed, pre-existing adaptive immune responses to the two most commonly used Cas9 proteins derived from *Staphylococcus pyogenes* (spCas9) and *Staphylococcus aureus* (saCas9) have been found in healthy adults (Charlesworth et al., 2019), confirming previous observations of immunity in the human population (Simhadri et al., 2018; Li et al., 2020). Also, viral mediated delivery mediates an indefinite expression of Cas enzymes, resulting in persistent recombination within the genome, and increasing the risk of endogenous genetic disruption and potential immune responses. Ongoing studies aiming to identify and engineer anti-CRISPRs, such as the DARPA Safe Genes program (https://www.darpa.mil/program/safe-genes), have already shown that one best path forward is the development of countermeasures that inhibit or reverse unwanted gene editing.

Improvements in our understanding of the biological effects of CRISPR on DNA, RNA, and protein should make it possible to increase the accuracy and long-term effectiveness of CRISPR systems, whilst mitigating the risks of gene editing, such as off-target effects, low efficacy rates, genotoxicity and immunogenicity. In the future, the search for chemical modifications, or bioengineering using directed evolution of Cas9 and gRNA tools will continue, in order to offer solutions: 1) to improve delivery to target cells, 2) to increase the specificity of interactions between the gRNA, DNA, and Cas nucleases, 3) to increase gRNA stability and 4) to reduce immunogenicity. These improvements will make it possible to expand the clinical use of CRISPR, to encompass all genetic disorders. Considering the interspecies differences in cellular targeting and gene editing efficiency, the validation of each gene editing protocol in small and large animals alongside human cells (such as those found in induced pluripotent stem cell derived organoids) will help provide key information on the disease target cells and determine therapeutic window according to disease developmental stages. The development of high-fidelity enzymes is tightly linked to the implementation of new methods, including machine learning modeling, for the design of CRISPR reagents and better prediction of beneficial outcomes (e.g., high on-target effects, low or absent off-targets) (Anzalone et al., 2020; Saha et al., 2021). Finally, an effective wide dissemination of all the data to the biomedical research community will be instrumental to a rapid development of adapted solutions and countermeasures to ensure efficient gene editing clinical treatment for most genetic diseases, broadly improving human health.

## References

[B1] Akil O. (2020). Dual and Triple AAV Delivery of Large Therapeutic Gene Sequences Into the Inner Ear. Hearing Res. 394, 107912. 10.1016/j.heares.2020.107912 32067799

[B2] Akil O. Dyka F. Calvet C. Emptoz A. Lahlou G. Nouaille S. (2019). Dual AAV-Mediated Gene Therapy Restores Hearing in a DFNB9 Mouse Model. Proc. Natl. Acad. Sci. USA. 116, 4496–4501. 10.1073/pnas.1817537116 30782832 PMC6410774

[B3] Akil O. Seal R. P. Burke K. Wang C. Alemi A. During M. (2012). Restoration of Hearing in the VGLUT3 Knockout Mouse Using Virally Mediated Gene Therapy. Neuron. 75, 283–293. 10.1016/j.neuron.2012.05.019 22841313 PMC3408581

[B4] Al-Moyed H. Cepeda A. P. Jung S. Moser T. Kügler S. Reisinger E. (2019). A Dual-AAV Approach Restores Fast Exocytosis and Partially Rescues Auditory Function in Deaf Otoferlin Knock-Out Mice. EMBO Mol. Med. 11, e9396. 10.15252/emmm.201809396 30509897 PMC6328916

[B5] An X. Zha D. (2020). Development of Nanoparticle Drug-Delivery Systems for the Inner Ear. Nanomedicine. 15, 1981–1993. 10.2217/nnm-2020-0198 32605499

[B6] Anzalone A. V. Koblan L. W. Liu D. R. (2020). Genome Editing With CRISPR-Cas Nucleases, Base Editors, Transposases and Prime Editors. Nat. Biotechnol. 38, 824–844. 10.1038/s41587-020-0561-9 32572269

[B7] Anzalone A. V. Randolph P. B. Davis J. R. Sousa A. A. Koblan L. W. Levy J. M. (2019). Search-and-Replace Genome Editing Without Double-Strand Breaks or Donor DNA. Nature. 576, 149–157. 10.1038/s41586-019-1711-4 31634902 PMC6907074

[B8] Askew C. Chien W. W. (2020). Adeno-Associated Virus Gene Replacement for Recessive Inner Ear Dysfunction: Progress and Challenges. Hearing Res. 394, 107947. 10.1016/j.heares.2020.107947 PMC7939749 32247629

[B9] Ayton L. N. Barnes N. Dagnelie G. Fujikado T. Goetz G. Hornig R. (2020). An Update on Retinal Prostheses. Clin. Neurophysiol. 131, 1383–1398. 10.1016/j.clinph.2019.11.029 31866339 PMC7198351

[B10] Azaiez H. Booth K. T. Ephraim S. S. Crone B. Black-Ziegelbein E. A. Marini R. J. (2018). Genomic Landscape and Mutational Signatures of Deafness-Associated Genes. Am. J. Hum. Genet. 103, 484–497. 10.1016/j.ajhg.2018.08.006 30245029 PMC6174355

[B11] Bakondi B. Lv W. Lu B. Jones M. K. Tsai Y. Kim K. J. (2016). *In Vivo* CRISPR/Cas9 Gene Editing Corrects Retinal Dystrophy in the S334ter-3 Rat Model of Autosomal Dominant Retinitis Pigmentosa. Mol. Ther. 24, 556–563. 10.1038/mt.2015.220 26666451 PMC4786918

[B12] Bennett J. Tanabe T. Sun D. Zeng Y. Kjeldbye H. Gouras P. (1996). Photoreceptor Cell Rescue in Retinal Degeneration (Rd) Mice by *In Vivo* Gene Therapy. Nat. Med. 2, 649–654. 10.1038/nm0696-649 8640555

[B13] Bonnet C. El-Amraoui A. (2012). Usher Syndrome (Sensorineural Deafness and Retinitis Pigmentosa). Curr. Opin. Neurol. 25, 42–49. 10.1097/wco.0b013e32834ef8b2 22185901

[B14] Botto C. Rucli M. Tekinsoy M. D. Pulman J. Sahel J.-A. Dalkara D. (2021). Early and Late Stage Gene Therapy Interventions for Inherited Retinal Degenerations. Prog. Retin. Eye Res., 100975. 10.1016/j.preteyeres.2021.100975 34058340

[B15] Bucher K. Rodríguez-Bocanegra E. Dauletbekov D. Fischer M. D. (2020). Immune Responses to Retinal Gene Therapy Using Adeno-Associated Viral Vectors - Implications for Treatment Success and Safety. Prog. Retin. Eye Res. 83, 100915. 10.1016/j.preteyeres.2020.100915 33069860

[B16] Buck T. M. Wijnholds J. (2020). Recombinant Adeno-Associated Viral Vectors (rAAV)-Vector Elements in Ocular Gene Therapy Clinical Trials and Transgene Expression and Bioactivity Assays. Int. J. Mol. Sci. 21, 4197. 10.3390/ijms21124197 PMC7352801 32545533

[B17] Byrne L. C. Day T. P. Visel M. Fortuny C. Dalkara D. Merigan W. H. (2020). *In Vivo* Directed Evolution of AAV in the Primate Retina. JCI Insight 5 (10), e135112. 10.1172/jci.insight.135112 PMC7259523 32271719

[B18] Cehajic-Kapetanovic J. Xue K. Martinez-Fernandez de la Camara C. Nanda A. Davies A. Wood L. J. (2020). Initial Results From a First-In-Human Gene Therapy Trial on X-Linked Retinitis Pigmentosa Caused by Mutations in RPGR. Nat. Med. 26, 354–359. 10.1038/s41591-020-0763-1 32094925 PMC7104347

[B19] Charlesworth C. T. Deshpande P. S. Dever D. P. Camarena J. Lemgart V. T. Cromer M. K. (2019). Identification of Preexisting Adaptive Immunity to Cas9 Proteins in Humans. Nat. Med. 25, 249–254. 10.1038/s41591-018-0326-x 30692695 PMC7199589

[B20] Chen G. Abdeen A. A. Wang Y. Shahi P. K. Robertson S. Xie R. (2019). A Biodegradable Nanocapsule Delivers a Cas9 Ribonucleoprotein Complex for *In Vivo* Genome Editing. Nat. Nanotechnol. 14, 974–980. 10.1038/s41565-019-0539-2 31501532 PMC6778035

[B21] Crane R. Conley S. M. Al-Ubaidi M. R. Naash M. I. (2021). Gene Therapy to the Retina and the Cochlea. Front. Neurosci. 15, 652215. 10.3389/fnins.2021.652215 33815052 PMC8010260

[B22] Cronin T. Vandenberghe L. H. Hantz P. Juttner J. Reimann A. Kacsó Á. E. (2014). Efficient Transduction and Optogenetic Stimulation of Retinal Bipolar Cells by a Synthetic Adeno‐Associated Virus Capsid and Promoter. EMBO Mol. Med. 6, 1175–1190. 10.15252/emmm.201404077 25092770 PMC4197864

[B23] de Bruijn S. E. Fadaie Z. Cremers F. P. M. Kremer H. Roosing S. (2021). The Impact of Modern Technologies on Molecular Diagnostic Success Rates, With a Focus on Inherited Retinal Dystrophy and Hearing Loss. Int. J. Mol. Sci. 22, 2943. 10.3390/ijms22062943 33799353 PMC7998853

[B24] Delmaghani S. El-Amraoui A. (2020). Inner Ear Gene Therapies Take off: Current Promises and Future Challenges. J. Clin. Med. 9, 2309–2336. 10.3390/jcm9072309 PMC7408650 32708116

[B25] Devoldere J. Peynshaert K. Dewitte H. Vanhove C. De Groef L. Moons L. (2019). Non-viral Delivery of Chemically Modified mRNA to the Retina: Subretinal Versus Intravitreal Administration. J. Controlled Release. 307, 315–330. 10.1016/j.jconrel.2019.06.042 31265881

[B26] Dryja T. P. McGee T. L. Hahn L. B. Cowley G. S. Olsson J. E. Reichel E. (1990). Mutations Within the Rhodopsin Gene in Patients With Autosomal Dominant Retinitis Pigmentosa. N. Engl. J. Med. 323, 1302–1307. 10.1056/nejm199011083231903 2215617

[B27] Dulla K. Slijkerman R. van Diepen H. C. Albert S. Dona M. Beumer W. (2021). Antisense Oligonucleotide-Based Treatment of Retinitis Pigmentosa Caused by USH2A Exon 13 Mutations. Mol. Ther. 29, 2441–2455. 10.1016/j.ymthe.2021.04.024 33895329 PMC8353187

[B28] Dulon D. Papal S. Patni P. Cortese M. Vincent P. F. Y. Tertrais M. (2018). Clarin-1 Gene Transfer Rescues Auditory Synaptopathy in Model of Usher Syndrome. J. Clin. Invest. 128, 3382–3401. 10.1172/jci94351 29985171 PMC6063508

[B29] Emptoz A. Michel V. Lelli A. Akil O. Boutet de Monvel J. Lahlou G. (2017). Local Gene Therapy Durably Restores Vestibular Function in a Mouse Model of Usher Syndrome Type 1G. Proc. Natl. Acad. Sci. U.S.A. 114 (36), 9695–9700. 10.1073/pnas.1708894114 28835534 PMC5594693

[B30] Fakhiri J. Landegger L. D. Grimm D. (2020). Breaking the Sound Barrier: Towards Next-Generation AAV Vectors for Gene Therapy of Hearing Disorders. Hearing Res., 108092. 10.1016/j.heares.2020.108092 33268240

[B31] Fry L. E. Peddle C. F. Barnard A. R. McClements M. E. MacLaren R. E. (2020). RNA Editing as a Therapeutic Approach for Retinal Gene Therapy Requiring Long Coding Sequences. Int. J. Mol. Sci. 21, 777. 10.3390/ijms21030777 PMC7037314 31991730

[B32] Gao X. Tao Y. Lamas V. Huang M. Yeh W.-H. Pan B. (2018). Treatment of Autosomal Dominant Hearing Loss by *In Vivo* Delivery of Genome Editing Agents. Nature. 553, 217–221. 10.1038/nature25164 29258297 PMC5784267

[B33] Garita-Hernandez M. Routet F. Guibbal L. Khabou H. Toualbi L. Riancho L. (2020). AAV-Mediated Gene Delivery to 3D Retinal Organoids Derived From Human Induced Pluripotent Stem Cells. Int. J. Mol. Sci. 21, E994. 10.3390/ijms21030994 32028585 PMC7036814

[B34] Gaudelli N. M. Komor A. C. Rees H. A. Packer M. S. Badran A. H. Bryson D. I. (2017). Programmable Base Editing of AT to GC in Genomic DNA Without DNA Cleavage. Nature. 551, 464–471. 10.1038/nature24644 29160308 PMC5726555

[B35] Géléoc G. G. S. El-Amraoui A. (2020). Disease Mechanisms and Gene Therapy for Usher Syndrome. Hearing Res. 394, 107932. 10.1016/j.heares.2020.107932 32199721

[B36] Geller A. M. Sieving P. A. Green D. G. (1992). Effect on Grating Identification of Sampling With Degenerate Arrays. J. Opt. Soc. Am. A. 9, 472–477. 10.1364/josaa.9.000472 1548555

[B37] Geller A. M. Sieving P. A. (1993). Assessment of Foveal Cone Photoreceptors in Stargardt's Macular Dystrophy Using a Small Dot Detection Task. Vis. Res. 33, 1509–1524. 10.1016/0042-6989(93)90144-l 8351823

[B38] Geng R. Omar A. Gopal S. R. Chen D. H.-C. Stepanyan R. Basch M. L. (2017). Modeling and Preventing Progressive Hearing Loss in Usher Syndrome III. Sci. Rep. 7, 13480. 10.1038/s41598-017-13620-9 29044151 PMC5647385

[B39] Giannelli S. G. Luoni M. Castoldi V. Massimino L. Cabassi T. Angeloni D. (2018). Cas9/sgRNA Selective Targeting of the P23H Rhodopsin Mutant Allele for Treating Retinitis Pigmentosa by Intravitreal AAV9.PHP.B-Based Delivery. Hum. Mol. Genet. 27, 761–779. 10.1093/hmg/ddx438 29281027

[B40] Gu X. Wang D. Xu Z. Wang J. Guo L. Chai R. (2021). Prevention of Acquired Sensorineural Hearing Loss in Mice by *In Vivo* Htra2 Gene Editing. Genome Biol. 22, 86. 10.1186/s13059-021-02311-4 33752742 PMC7983387

[B41] György B. Meijer E. J. Ivanchenko M. V. Tenneson K. Emond F. Hanlon K. S. (2019a). Gene Transfer With AAV9-PHP.B Rescues Hearing in a Mouse Model of Usher Syndrome 3A and Transduces Hair Cells in a Non-Human Primate. Mol. Ther. - Methods Clin. Development. 13, 1–13. 10.1016/j.omtm.2018.11.003 PMC6297893 30581889

[B42] György B. Nist-Lund C. Pan B. Asai Y. Karavitaki K. D. Kleinstiver B. P. (2019b). Allele-specific Gene Editing Prevents Deafness in a Model of Dominant Progressive Hearing Loss. Nat. Med. 25, 1123–1130. 10.1038/s41591-019-0500-9 31270503 PMC6802276

[B43] Hirakawa M. P. Krishnakumar R. Timlin J. A. Carney J. P. Butler K. S. (2020). Gene Editing and CRISPR in the Clinic: Current and Future Perspectives. Biosci. Rep. 40, BSR20200127. 10.1042/BSR20200127 32207531 PMC7146048

[B44] Holmgaard A. B. Askou A. L. Jensen E. G. Alsing S. Bak R. O. Mikkelsen J. G. (2021). Targeted Knockout of the Vegfa Gene in the Retina by Subretinal Injection of RNP Complexes Containing Cas9 Protein and Modified sgRNAs. Mol. Ther. 29, 191–207. 10.1016/j.ymthe.2020.09.032 33022212 PMC7791085

[B45] Huang T. P. Newby G. A. Liu D. R. (2021). Precision Genome Editing Using Cytosine and Adenine Base Editors in Mammalian Cells. Nat. Protoc. 16, 1089–1128. 10.1038/s41596-020-00450-9 33462442

[B46] Husseman J. Raphael Y. (2009). Gene Therapy in the Inner Ear Using Adenovirus Vectors. Adv. Otorhinolaryngol. 66, 37–51. 10.1159/000218206 19494571 PMC4464776

[B47] Iizuka T. Kamiya K. Gotoh S. Sugitani Y. Suzuki M. Noda T. (2015). Perinatal Gjb2 Gene Transfer Rescues Hearing in a Mouse Model of Hereditary Deafness. Hum. Mol. Genet. 24, 3651–3661. 10.1093/hmg/ddv109 25801282

[B48] Ingham N. J. Pearson S. A. Vancollie V. E. Rook V. Lewis M. A. Chen J. (2019). Mouse Screen Reveals Multiple New Genes Underlying Mouse and Human Hearing Loss. Plos Biol. 17, e3000194. 10.1371/journal.pbio.3000194 30973865 PMC6459510

[B49] Isgrig K. McDougald D. S. Zhu J. Wang H. J. Bennett J. Chien W. W. (2019). AAV2.7m8 Is a Powerful Viral Vector for Inner Ear Gene Therapy. Nat. Commun. 10, 427. 10.1038/s41467-018-08243-1 30683875 PMC6347594

[B50] Isgrig K. Shteamer J. W. Belyantseva I. A. Drummond M. C. Fitzgerald T. S. Vijayakumar S. (2017). Gene Therapy Restores Balance and Auditory Functions in a Mouse Model of Usher Syndrome. Mol. Ther. 25, 780–791. 10.1016/j.ymthe.2017.01.007 28254438 PMC5363211

[B51] Jüttner J. Szabo A. Gross-Scherf B. Morikawa R. K. Rompani S. B. Hantz P. (2019). Targeting Neuronal and Glial Cell Types With Synthetic Promoter AAVs in Mice, Non-Human Primates and Humans. Nat. Neurosci. 22, 1345–1356. 10.1038/s41593-019-0431-2 31285614

[B52] Kim E. Koo T. Park S. W. Kim D. Kim K. Cho H.-Y. (2017a). *In Vivo* genome Editing With a Small Cas9 Orthologue Derived From Campylobacter Jejuni. Nat. Commun. 8, 14500. 10.1038/ncomms14500 28220790 PMC5473640

[B53] Kim K. Park S. W. Kim J. H. Lee S. H. Kim D. Koo T. (2017b). Genome Surgery Using Cas9 Ribonucleoproteins for the Treatment of Age-Related Macular Degeneration. Genome Res. 27, 419–426. 10.1101/gr.219089.116 28209587 PMC5340969

[B54] Kleinlogel S. Vogl C. Jeschke M. Neef J. Moser T. (2020). Emerging Approaches for Restoration of Hearing and Vision. Physiol. Rev. 100, 1467–1525. 10.1152/physrev.00035.2019 32191560

[B55] Komor A. C. Kim Y. B. Packer M. S. Zuris J. A. Liu D. R. (2016). Programmable Editing of a Target Base in Genomic DNA Without Double-Stranded DNA Cleavage. Nature. 533, 420–424. 10.1038/nature17946 27096365 PMC4873371

[B56] Köppl C. Manley G. A. (2019). A Functional Perspective on the Evolution of the Cochlea. Cold Spring Harb Perspect. Med. 9, a033241. 10.1101/cshperspect.a033241 30181353 PMC6546037

[B57] Kremer H. (2019). Hereditary Hearing Loss; about the Known and the Unknown. Hearing Res. 376, 58–68. 10.1016/j.heares.2019.01.003 30665849

[B58] Lan Y. Tao Y. Wang Y. Ke J. Yang Q. Liu X. (2020). Recent Development of AAV-Based Gene Therapies for Inner Ear Disorders. Gene Ther. 27 (7-8), 329–337. 10.1038/s41434-020-0155-7 32424232 PMC7445886

[B59] Landegger L. D. Pan B. Askew C. Wassmer S. J. Gluck S. D. Galvin A. (2017). A Synthetic AAV Vector Enables Safe and Efficient Gene Transfer to the Mammalian Inner Ear. Nat. Biotechnol. 35, 280–284. 10.1038/nbt.3781 28165475 PMC5340646

[B60] Landrum M. J. Lee J. M. Benson M. Brown G. Chao C. Chitipiralla S. (2016). ClinVar: Public Archive of Interpretations of Clinically Relevant Variants. Nucleic Acids Res. 44, D862–D868. 10.1093/nar/gkv1222 26582918 PMC4702865

[B61] Latella M. C. Di Salvo M. T. Cocchiarella F. Benati D. Grisendi G. Comitato A. (2016). *In Vivo* Editing of the Human Mutant Rhodopsin Gene by Electroporation of Plasmid-Based CRISPR/Cas9 in the Mouse Retina. Mol. Ther. - Nucleic Acids. 5, e389. 10.1038/mtna.2016.92 27874856 PMC5155324

[B62] Leis J. A. Rutka J. A. Gold W. L. (2015). Aminoglycoside-Induced Ototoxicity. CMAJ. 187, E52. 10.1503/cmaj.140339 25225217 PMC4284193

[B63] Lentz J. J. Pan B. Ponnath A. Tran C. M. Nist-Lund C. Galvin A. (2020). Direct Delivery of Antisense Oligonucleotides to the Middle and Inner Ear Improves Hearing and Balance in Usher Mice. Mol. Ther. 28, 2662–2676. 10.1016/j.ymthe.2020.08.002 32818431 PMC7704764

[B64] Levy J. M. Yeh W.-H. Pendse N. Davis J. R. Hennessey E. Butcher R. (2020). Cytosine and Adenine Base Editing of the Brain, Liver, Retina, Heart and Skeletal Muscle of Mice via Adeno-Associated Viruses. Nat. Biomed. Eng. 4, 97–110. 10.1038/s41551-019-0501-5 31937940 PMC6980783

[B65] Li A. Tanner M. R. Lee C. M. Hurley A. E. De Giorgi M. Jarrett K. E. (2020). AAV-CRISPR Gene Editing Is Negated by Pre-Existing Immunity to Cas9. Mol. Ther. 28, 1432–1441. 10.1016/j.ymthe.2020.04.017 32348718 PMC7264438

[B66] Li L. Chao T. Brant J. O'Malley B. Jr. Tsourkas A. Li D. (2017). Advances in Nano-Based Inner Ear Delivery Systems for the Treatment of Sensorineural Hearing Loss. Adv. Drug Deliv. Rev. 108, 2–12. 10.1016/j.addr.2016.01.004 26796230 PMC4940320

[B67] Li P. Kleinstiver B. P. Leon M. Y. Prew M. S. Navarro-Gomez D. Greenwald S. H. (2018). Allele-Specific CRISPR-Cas9 Genome Editing of the Single-Base P23H Mutation for Rhodopsin-Associated Dominant Retinitis Pigmentosa. CRISPR J. 1, 55–64. 10.1089/crispr.2017.0009 31021187 PMC6319323

[B68] Maddalena A. Tornabene P. Tiberi P. Minopoli R. Manfredi A. Mutarelli M. (2018). Triple Vectors Expand AAV Transfer Capacity in the Retina. Mol. Ther. 26, 524–541. 10.1016/j.ymthe.2017.11.019 29292161 PMC5835116

[B69] Maeda Y. Sheffield A. M. Smith R. J. H. (2009). Therapeutic Regulation of Gene Expression in the Inner Ear Using RNA Interference. Adv. Otorhinolaryngol. 66, 13–36. 10.1159/000218205 19494570 PMC2867253

[B70] Maeder M. L. Stefanidakis M. Wilson C. J. Baral R. Barrera L. A. Bounoutas G. S. (2019). Development of a Gene-Editing Approach to Restore Vision Loss in Leber Congenital Amaurosis Type 10. Nat. Med. 25, 229–233. 10.1038/s41591-018-0327-9 30664785

[B71] Maguire A. M. Bennett J. Aleman E. M. Leroy B. P. Aleman T. S. (2021). Clinical Perspective: Treating RPE65-Associated Retinal Dystrophy. Mol. Ther. 29, 442–463. 10.1016/j.ymthe.2020.11.029 33278565 PMC7854308

[B72] Malhotra H. Barnes C. L. Calvert P. D. (2021). Functional Compartmentalization of Photoreceptor Neurons. Pflugers Arch. - Eur. J. Physiol. 473, 1493–1516. 10.1007/s00424-021-02558-7 33880652 PMC8690575

[B73] Manji S. S. M. Miller K. A. Williams L. H. Dahl H.-H. M. (2012). Identification of Three Novel Hearing Loss Mouse Strains With Mutations in the Tmc1 Gene. Am. J. Pathol. 180, 1560–1569. 10.1016/j.ajpath.2011.12.034 22330676

[B74] McCullough K. T. Boye S. L. Fajardo D. Calabro K. Peterson J. J. Strang C. E. (2019). Somatic Gene Editing of GUCY2D by AAV-CRISPR/Cas9 Alters Retinal Structure and Function in Mouse and Macaque. Hum. Gene Ther. 30, 571–589. 10.1089/hum.2018.193 30358434 PMC6534089

[B75] Mével M. Bouzelha M. Leray A. Pacouret S. Guilbaud M. Penaud-Budloo M. (2019). Chemical Modification of the Adeno-Associated Virus Capsid to Improve Gene Delivery. Chem. Sci. 11, 1122–1131. 10.1039/c9sc04189c 34084369 PMC8145868

[B76] Mittal R. Pena S. A. Zhu A. Eshraghi N. Fesharaki A. Horesh E. J. (2019). Nanoparticle-based Drug Delivery in the Inner Ear: Current Challenges, Limitations and Opportunities. Artif. Cell Nanomedicine, Biotechnol. 47, 1312–1320. 10.1080/21691401.2019.1573182 30987439

[B77] Moreno A. M. Fu X. Zhu J. Katrekar D. Shih Y.-R. V. Marlett J. (2018). *In Situ* Gene Therapy via AAV-CRISPR-Cas9-Mediated Targeted Gene Regulation. Mol. Ther. 26, 1818–1827. 10.1016/j.ymthe.2018.04.017 29754775 PMC6035733

[B78] Müller U. Barr-Gillespie P. G. (2015). New Treatment Options for Hearing Loss. Nat. Rev. Drug Discov. 14, 346–365. 10.1038/nrd4533 25792261

[B79] Niggemann P. György B. Chen Z.-Y. (2020). Genome and Base Editing for Genetic Hearing Loss. Hearing Res. 394, 107958. 10.1016/j.heares.2020.107958 PMC7415640 32334889

[B80] Nist-Lund C. A. Pan B. Patterson A. Asai Y. Chen T. Zhou W. (2019). Improved TMC1 Gene Therapy Restores Hearing and Balance in Mice With Genetic Inner Ear Disorders. Nat. Commun. 10, 236. 10.1038/s41467-018-08264-w 30670701 PMC6342993

[B81] Nourbakhsh A. Colbert B. M. Nisenbaum E. El-Amraoui A. Dykxhoorn D. M. Koehler K. R. (2021). Stem Cells and Gene Therapy in Progressive Hearing Loss: the State of the Art. J. Assoc. Res. Otolaryngol. 22, 95–105. 10.1007/s10162-020-00781-0 33507440 PMC7943682

[B82] Nyberg S. Abbott N. J. Shi X. Steyger P. S. Dabdoub A. (2019). Delivery of Therapeutics to the Inner Ear: The Challenge of the Blood-Labyrinth Barrier. Sci. Transl Med. 11, eaao0935. 10.1126/scitranslmed.aao0935 30842313 PMC6488020

[B83] Omichi R. Yoshimura H. Shibata S. B. Vandenberghe L. H. Smith R. J. H. (2020). Hair Cell Transduction Efficiency of Single- and Dual-AAV Serotypes in Adult Murine Cochleae. Mol. Ther. - Methods Clin. Development. 17, 1167–1177. 10.1016/j.omtm.2020.05.007 PMC7270144 32518805

[B84] Pan B. Askew C. Galvin A. Heman-Ackah S. Asai Y. Indzhykulian A. A. (2017). Gene Therapy Restores Auditory and Vestibular Function in a Mouse Model of Usher Syndrome Type 1c. Nat. Biotechnol. 35, 264–272. 10.1038/nbt.3801 28165476 PMC5340578

[B85] Patel S. Ryals R. C. Weller K. K. Pennesi M. E. Sahay G. (2019). Lipid Nanoparticles for Delivery of Messenger RNA to the Back of the Eye. J. Controlled Release. 303, 91–100. 10.1016/j.jconrel.2019.04.015 PMC6579630 30986436

[B86] Pavlou M. Schon C. Occelli L. M. Rossi A. Meumann N. Boyd R. F. (2021). Novel AAV Capsids for Intravitreal Gene Therapy of Photoreceptor Disorders. EMBO Mol. Med. 13, e13392. 10.15252/emmm.202013392 33616280 PMC8033523

[B87] Perrault I. Delphin N. Hanein S. Gerber S. Dufier J.-L. Roche O. (2007). Spectrum of NPHP6/CEP290 Mutations in Leber Congenital Amaurosis and Delineation of the Associated Phenotype. Hum. Mutat. 28, 416. 10.1002/humu.9485 17345604

[B88] Petrs-Silva H. Dinculescu A. Li Q. Deng W.-T. Pang J.-j. Min S.-H. (2011). Novel Properties of Tyrosine-Mutant AAV2 Vectors in the Mouse Retina. Mol. Ther. 19, 293–301. 10.1038/mt.2010.234 21045809 PMC3034844

[B89] Planul A. Dalkara D. (2017). Vectors and Gene Delivery to the Retina. Annu. Rev. Vis. Sci. 3, 121–140. 10.1146/annurev-vision-102016-061413 28937950

[B90] Puppo A. Cesi G. Marrocco E. Piccolo P. Jacca S. Shayakhmetov D. M. (2014). Retinal Transduction Profiles by High-Capacity Viral Vectors. Gene Ther. 21, 855–865. 10.1038/gt.2014.57 24989814 PMC4193889

[B91] Ran F. A. Cong L. Yan W. X. Scott D. A. Gootenberg J. S. Kriz A. J. (2015). *In Vivo* genome Editing Using *Staphylococcus aureus* Cas9. Nature. 520, 186–191. 10.1038/nature14299 25830891 PMC4393360

[B92] Rees H. A. Liu D. R. (2018). Base Editing: Precision Chemistry on the Genome and Transcriptome of Living Cells. Nat. Rev. Genet. 19, 770–788. 10.1038/s41576-018-0059-1 30323312 PMC6535181

[B93] Reisinger E. (2019). Dual-AAV Delivery of Large Gene Sequences to the Inner Ear. Hear. Res. 394, 107857. 10.1016/j.heares.2019.107857 31810595

[B94] Rossi E. A. Chung M. Dubra A. Hunter J. J. Merigan W. H. Williams D. R. (2011). Imaging Retinal Mosaics in the Living Eye. Eye. 25, 301–308. 10.1038/eye.2010.221 21390064 PMC3178316

[B95] Russell S. Bennett J. Wellman J. A. Chung D. C. Yu Z.-F. Tillman A. (2017). Efficacy and Safety of Voretigene Neparvovec (AAV2-hRPE65v2) in Patients With RPE65 -mediated Inherited Retinal Dystrophy: a Randomised, Controlled, Open-Label, Phase 3 Trial. The Lancet. 390, 849–860. 10.1016/s0140-6736(17)31868-8 PMC5726391 28712537

[B96] Sacheli R. Delacroix L. Vandenackerveken P. Nguyen L. Malgrange B. (2013). Gene Transfer in Inner Ear Cells: a Challenging Race. Gene Ther. 20, 237–247. 10.1038/gt.2012.51 22739386

[B97] Safieddine S. El-Amraoui A. Petit C. (2012). The Auditory Hair Cell Ribbon Synapse: from Assembly to Function. Annu. Rev. Neurosci. 35, 509–528. 10.1146/annurev-neuro-061010-113705 22715884

[B98] Saha K. Sontheimer E. J. Sontheimer E. J. Brooks P. J. Dwinell M. R. Gersbach C. A. (2021). The NIH Somatic Cell Genome Editing Program. Nature. 592, 195–204. 10.1038/s41586-021-03191-1 33828315 PMC8026397

[B99] Sahel J.-A. Boulanger-Scemama E. Pagot C. Arleo A. Galluppi F. Martel J. N. (2021). Partial Recovery of Visual Function in a Blind Patient after Optogenetic Therapy. Nat. Med. 27, 1223–1229. 10.1038/s41591-021-01351-4 34031601

[B100] Sahu B. Chug I. Khanna H. (2021). The Ocular Gene Delivery Landscape. Biomolecules. 11, 1135. 10.3390/biom11081135 34439800 PMC8394578

[B101] Sanjurjo-Soriano C. Erkilic N. Baux D. Mamaeva D. Hamel C. P. Meunier I. (2020). Genome Editing in Patient iPSCs Corrects the Most Prevalent USH2A Mutations and Reveals Intriguing Mutant mRNA Expression Profiles. Mol. Ther. - Methods Clin. Development. 17, 156–173. 10.1016/j.omtm.2019.11.016 PMC6938853 31909088

[B102] Scholl H. P. Strauss R. W. Singh M. S. Dalkara D. Roska B. Picaud S. (2016). Emerging Therapies for Inherited Retinal Degeneration. Sci. Transl Med. 8, 368rv6. 10.1126/scitranslmed.aaf2838 27928030

[B103] Shearer A. E. Hildebrand M. S. Smith R. J. H. (1993). “Hereditary Hearing Loss and Deafness Overview,” in GeneReviews((R)). Editors Adam M. P. Ardinger H. H. Pagon R. A. Wallace S. E. Bean L. J. H. Mirzaa G. (Seattle (WA)).

[B104] Shi F. Cheng Y.-f. Wang X. L. Edge A. S. B. (2010). β-Catenin Up-Regulates Atoh1 Expression in Neural Progenitor Cells by Interaction With an Atoh1 3′ Enhancer. J. Biol. Chem. 285, 392–400. 10.1074/jbc.m109.059055 19864427 PMC2804186

[B105] Shu Y. Tao Y. Li W. Shen J. Wang Z. Chen Z. Y. (2016). Adenovirus Vectors Target Several Cell Subtypes of Mammalian Inner Ear *In Vivo* . Neural Plast. 2016, 9409846. 10.1155/2016/9409846 28116172 PMC5225386

[B106] Simhadri V. L. McGill J. McMahon S. Wang J. Jiang H. Sauna Z. E. (2018). Prevalence of Pre-Existing Antibodies to CRISPR-Associated Nuclease Cas9 in the USA Population. Mol. Ther. - Methods Clin. Development. 10, 105–112. 10.1016/j.omtm.2018.06.006 PMC6070699 30073181

[B107] Stojkovic M. Han D. Jeong M. Stojkovic P. Stankovic K. M. (2021). Human Induced Pluripotent Stem Cells and CRISPR/Cas-Mediated Targeted Genome Editing: Platforms to Tackle Sensorineural Hearing Loss. Stem Cells. 39, 673–696. 10.1002/stem.3353 33586253

[B108] Suh S. Choi E. H. Leinonen H. Foik A. T. Newby G. A. Yeh W.-H. (2021). Restoration of Visual Function in Adult Mice With an Inherited Retinal Disease via Adenine Base Editing. Nat. Biomed. Eng. 5, 169–178. 10.1038/s41551-020-00632-6 33077938 PMC7885272

[B109] Sung C. H. Davenport C. M. Hennessey J. C. Maumenee I. H. Jacobson S. G. Heckenlively J. R. (1991). Rhodopsin Mutations in Autosomal Dominant Retinitis Pigmentosa. Proc. Natl. Acad. Sci. 88, 6481–6485. 10.1073/pnas.88.15.6481 1862076 PMC52109

[B110] Suzuki K. Izpisua Belmonte J. C. (2018). *In Vivo* genome Editing via the HITI Method as a Tool for Gene Therapy. J. Hum. Genet. 63, 157–164. 10.1038/s10038-017-0352-4 29215090

[B111] Suzuki K. Tsunekawa Y. Hernandez-Benitez R. Wu J. Zhu J. Kim E. J. (2016). *In Vivo* genome Editing via CRISPR/Cas9 Mediated Homology-Independent Targeted Integration. Nature. 540, 144–149. 10.1038/nature20565 27851729 PMC5331785

[B112] Taiber S. Cohen R. Yizhar-Barnea O. Sprinzak D. Holt J. R. Avraham K. B. (2021). Neonatal AAV Gene Therapy Rescues Hearing in a Mouse Model of SYNE4 Deafness. EMBO Mol. Med. 13, e13259. 10.15252/emmm.202013259 33350593 PMC7863404

[B113] Thompson D. A. Iannaccone A. Ali R. R. Arshavsky V. Y. Audo I. Bainbridge J. W. B. (2020). Advancing Clinical Trials for Inherited Retinal Diseases: Recommendations From the Second Monaciano Symposium. Trans. Vis. Sci. Tech. 9, 2. 10.1167/tvst.9.7.2 PMC7414644 32832209

[B114] Toualbi L. Toms M. Moosajee M. (2021). The Landscape of Non-Viral Gene Augmentation Strategies for Inherited Retinal Diseases. Int. J. Mol. Sci. 22, 2318. 10.3390/ijms22052318 33652562 PMC7956638

[B115] Trapani I. Tornabene P. Auricchio A. (2020). Large Gene Delivery to the Retina With AAV Vectors: Are We There yet? Gene Ther. 28, 220–222. 10.1038/s41434-020-0174-4 32661283

[B116] Vaerman J. P. Heremans J. F. (1968). Effect of Neuraminidase and Acidification on Complement-Fixing Properties of Human IgA and IgG. Int. Arch. Allergy Immunol. 34, 49–52. 10.1159/000230093 4174443

[B117] Valentini C. Szeto B. Kysar J. W. Lalwani A. K. (2020). Inner Ear Gene Delivery: Vectors and Routes. Hearing, Balance Commun. 18, 278–285. 10.1080/21695717.2020.1807261 33604229 PMC7888570

[B118] Vreugde S. Erven A. Kros C. J. Marcotti W. Fuchs H. Kurima K. (2002). Beethoven, a Mouse Model for Dominant, Progressive Hearing Loss DFNA36. Nat. Genet. 30, 257–258. 10.1038/ng848 11850623

[B119] Wang D. Tai P. W. L. Gao G. (2019). Adeno-Associated Virus Vector as a Platform for Gene Therapy Delivery. Nat. Rev. Drug Discov. 18, 358–378. 10.1038/s41573-019-0012-9 30710128 PMC6927556

[B120] Wu J. Solanes P. Nist-Lund C. Spataro S. Shubina-Oleinik O. Marcovich I. (2021). Single and Dual Vector Gene Therapy With AAV9-PHP.B Rescues Hearing in Tmc1 Mutant Mice. Mol. Ther. 29, 973–988. 10.1016/j.ymthe.2020.11.016 33212302 PMC7934577

[B121] Wu S.-S. Li Q.-C. Yin C.-Q. Xue W. Song C.-Q. (2020). Advances in CRISPR/Cas-based Gene Therapy in Human Genetic Diseases. Theranostics. 10, 4374–4382. 10.7150/thno.43360 32292501 PMC7150498

[B122] Yeh W.-H. Chiang H. Rees H. A. Edge A. S. B. Liu D. R. (2018). *In Vivo* Base Editing of Post-Mitotic Sensory Cells. Nat. Commun. 9, 2184. 10.1038/s41467-018-04580-3 29872041 PMC5988727

[B123] Yeh W. H. Shubina-Oleinik O. Levy J. M. Pan B. Newby G. A. Wornow M. (2020). *In Vivo* Base Editing Restores Sensory Transduction and Transiently Improves Auditory Function in a Mouse Model of Recessive Deafness. Sci. Transl Med. 12, eaay9101. 10.1126/scitranslmed.aay9101 32493795 PMC8167884

[B124] Yu W. Mookherjee S. Chaitankar V. Hiriyanna S. Kim J.-W. Brooks M. (2017). Nrl Knockdown by AAV-Delivered CRISPR/Cas9 Prevents Retinal Degeneration in Mice. Nat. Commun. 8, 14716. 10.1038/ncomms14716 28291770 PMC5355895

[B125] Yu W. Wu Z. (2021). Ocular Delivery of CRISPR/Cas Genome Editing Components for Treatment of Eye Diseases. Adv. Drug Deliv. Rev. 168, 181–195. 10.1016/j.addr.2020.06.011 32603815

[B126] Zhang D. Hussain A. Manghwar H. Xie K. Xie S. Zhao S. (2020a). Genome Editing With the CRISPR‐Cas System: an Art, Ethics and Global Regulatory Perspective. Plant Biotechnol. J. 18, 1651–1669. 10.1111/pbi.13383 32271968 PMC7336378

[B127] Zhang L. Wu X. Lin X. (2020b). Gene Therapy for Genetic Mutations Affecting Non-Sensory Cells in the Cochlea. Hearing Res. 394, 107858. 10.1016/j.heares.2019.107858 31791650

[B128] Zhao Y. Wang D. Zong L. Zhao F. Guan L. Zhang P. (2014). A Novel DFNA36 Mutation in TMC1 Orthologous to the Beethoven (Bth) Mouse Associated With Autosomal Dominant Hearing Loss in a Chinese Family. PLoS One. 9, e97064. 10.1371/journal.pone.0097064 24827932 PMC4020765

[B129] Zine A. Messat Y. Fritzsch B. (2021). A Human Induced Pluripotent Stem Cell-Based Modular Platform to Challenge Sensorineural Hearing Loss. Stem Cells. 39, 697–706. 10.1002/stem.3346 33522002 PMC8359331

[B130] Zinn E. Pacouret S. Khaychuk V. Turunen H. T. Carvalho L. S. Andres-Mateos E. (2015). In Silico Reconstruction of the Viral Evolutionary Lineage Yields a Potent Gene Therapy Vector. Cel Rep. 12, 1056–1068. 10.1016/j.celrep.2015.07.019 PMC4536165 26235624

[B131] Zuris J. A. Thompson D. B. Shu Y. Guilinger J. P. Bessen J. L. Hu J. H. (2015). Cationic Lipid-Mediated Delivery of Proteins Enables Efficient Protein-Based Genome Editing *In Vitro* and *In Vivo* . Nat. Biotechnol. 33, 73–80. 10.1038/nbt.3081 25357182 PMC4289409

